# Rapid photoinduced charge injection into covalent polyoxometalate–bodipy conjugates†
†Electronic supplementary information (ESI) available: General methods, reaction procedures, characterization of the hybrids, differential pulse voltammograms, spectroelectrochemical measurements and transient absorption spectra. See DOI: 10.1039/c8sc00862k


**DOI:** 10.1039/c8sc00862k

**Published:** 2018-06-01

**Authors:** Fiona A. Black, Aurélie Jacquart, Georgios Toupalas, Sandra Alves, Anna Proust, Ian P. Clark, Elizabeth A. Gibson, Guillaume Izzet

**Affiliations:** a Chemistry: School of Natural and Environmental Science , Newcastle University , Newcastle upon Tyne , NE1 7RU , UK . Email: Elizabeth.gibson@ncl.ac.uk; b Sorbonne Université , CNRS , Institut Parisien de Chimie Moléculaire , IPCM , 4 Place Jussieu , F-75005 Paris , France . Email: guillaume.izzet@sorbonne-universite.fr; c Central Laser Facility, Research Complex at Harwell , Science and Technology Facilities Council , Rutherford Appleton Laboratory , Harwell Oxford , Didcot , Oxfordshire OX11 0QX , UK

## Abstract

Controlled design of photoactive hybrids would provide highly active materials for solar energy conversion and photo(electro) catalysis. We describe the kinetics of photoinduced electron transfer in a series of photoactive hybrids based on Keggin-type polyoxometalates (POMs) covalently grafted to bodipy photosensitizers. We show how the electronic properties and corresponding dynamics of these hybrids can be readily tuned by varying the POM metal ion, the anchoring functionalization and the spacer length. Ultrafast visible and IR transient absorption spectroscopy, supported by spectroelectrochemical measurements, reveals that photoinduced electron transfer from the bodipy chromophore to the organosilyl POM derivative occurs as rapidly as *τ* = 54 ps to generate a long-lived (*τ* = 4.8 ns) charge-separated (CS) state, making this system appropriate for applications in photoelectrochemical devices.

## Abstract 2

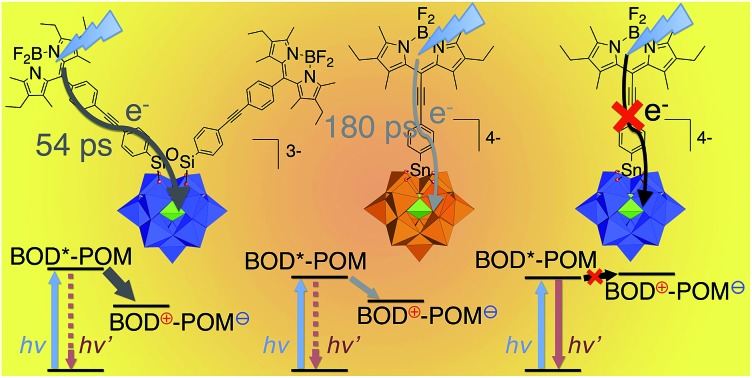
A series of redox tunable polyoxometalate–bodipy conjugates display variable charge transfer dynamics occuring down to 54 ps.

## Introduction

The direct generation of chemical fuels from sunlight is a major scientific challenge for the development of a sustainable economy and production of hydrogen through water splitting. In green plants, photosynthesis operates in four steps: (1) light collection, (2) charge separation, (3) charge accumulation and (4) conversion to chemical fuel. Charge accumulation is a key step as most of the redox reactions for fuel generation such as H_2_ production from water are multi-electronic processes.^1^ While various noble-metal-based complexes (*e.g.* Pd, Pt…) have been widely employed as reduction catalysts in artificial photosynthetic systems, polyoxometalates have recently emerged in this field owing to their electron reservoir abilities and the activity of their reduced forms in the hydrogen evolution reaction.^2^–^4^ POMs form a remarkable class of well-defined molecular nano-scale oxoclusters of the early transition metals with an unmatched diversity of structures and properties.^5^ Their implementation in artificial photosynthetic devices requires their association with a visible-range antenna since POMs only absorb in the UV region of the solar spectrum. Owing to their anionic nature, POMs have mainly been associated with photosensitizer (PS) through electrostatic interactions.^6^–^11^ By comparison, few covalent organic–inorganic POM-based hybrids have been developed.^12^–^17^ While the covalent functionalization of POMs is synthetically more demanding, this approach enables fine control between the different subunits of the system, which is required for tuning the kinetics of photoinduced electron transfer between the excited chromophore and the POM.^3^

We previously reported the synthesis and photophysical properties of different POM–PS conjugates.^18^–^23^ Among them heteroleptic carbocyclometalated iridium(iii)-POM dyads offered the most promising photophysical performance.^23^ In these compounds, photoinduced charge-separated excited states of various lifetimes (ranging from nanoseconds to hundreds of nanoseconds, the longest values reported for covalently bonded photosensitized POMs) were observed by transient absorption spectroscopy. Furthermore, in the presence of a sacrificial electron donor (triethylamine) and a proton source (acetic acid), the system is capable of photo-accumulating two electrons on the POM and produces hydrogen.^20^ However, the POM-Ir(iii) system suffered from the presence of a noble metal (Ir) and the low stability of the iridium complex in the presence of strong acid, which is necessary to improve the electron reservoir properties of the POM.

**Scheme 1:**
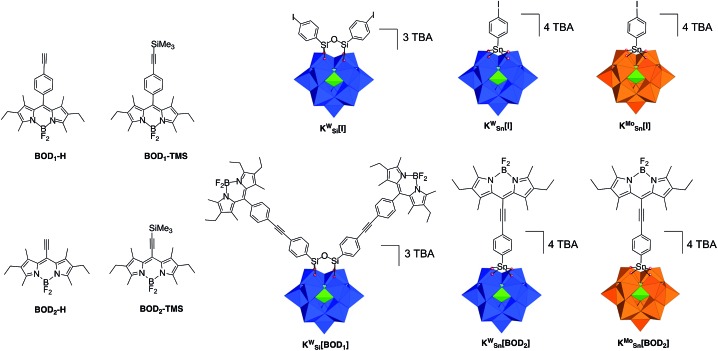
Molecular drawings of the bodipy reference compounds (**BOD_1_-H**, **BOD_1_-TMS**, **BOD_2_-H**, **BOD_2_-TMS**), the iodoaryl-terminated POM-based platforms (**K_Si_^W^[I]**, **K_Sn_^W^[I]**, **K_Sn_^Mo^[I]**) and the POM–bodipy conjugates described in this study (**K_Si_^W^[BOD_1_]**, **K_Sn_^W^[BOD_2_]**, **K_Sn_^Mo^[BOD_2_]**). Color code: WO_6_ octahedra, blue; MoO_6_ octahedra, blue; PO_4_ tetrahedra, green.

Furthermore, in order to incorporate the POM–PS hybrids into a photocathode, the functionalization of the PS by an appropriate anchoring group (carboxylic acid, phosphonate…) is a required step, which has been very scarcely developed with carbocyclometalated iridium(iii) complexes.^24^ In this context, bodipy fluorophores have been identified as adequate candidates because of their tunable photophysical properties, high chemical stability, easy and versatile chemical modification such as the addition of a grafting function.^25^ Here, we present the first examples of Keggin-type POMs that are covalently bound to one or two bodipy units *via* organic tethers of different lengths (Scheme 1). The series of three hybrids display distinct redox behaviour both on the POM and on the bodipy units. The photophysical properties of these hybrids are reported and discussed on the basis of the required kinetics that should be achieved for their implementation in a photoelectrocatalytic device.

## Results and discussion

### Synthesis of the different POM–bodipy conjugates

We previously developed three Keggin-based polyoxometalate hybrid platforms bearing one or two remote iodo aryl functions. These hybrids vary according to the nature of the metal ion (polyoxotungstate *vs.* polyoxomolybdate) and the nature of the primary functionalization (silyl *vs.* tin).^21^,^26^,^27^ The organosilyl POM derivative (noted **K_Si_^W^[I]**‡
‡Acronyms used for the POM hybrids: K or D refers to the Keggin or the Dawson-type anion, Mo or W as superscript indicates the metal of the POM backbone, Si, or Sn, as subscript relates to the primary functionalization and the term in brackets corresponds to the remote organic moieties.) is more easily reducible than its organotin counterparts due to a lower charge. Furthermore, the reduction processes are more accessible on the polyoxomolybdate organotin derivative (noted **K_Sn_^Mo^[I]**) compared to its isocharged tungstate analogue (noted **K_Sn_^W^[I]**), as classically observed for polyoxometalates.^27^,^28^ The electron acceptor abilities of these hybrids thus follow the trend: **K_Si_^W^[I]** > **K_Sn_^Mo^[I]** > **K_Sn_^W^[I]**.^3^

In a first attempt we tried to couple the organosilyl polyoxotungstate platform **K_Si_^W^[I]** (displaying the best electron accepting properties) with both alkyne terminated bodipy **BOD_1_-H** or **BOD_2_-H** in order to investigate the effect of the spacer length on the photophysical properties using the same Keggin-based platform. However, while **K_Si_^W^[BOD_1_]** turned out to be stable, the hybrid resulting from the coupling between **K_Si_^W^[I]** and **BOD_2_-H** unexpectedly slightly decomposed upon precipitation. Consequently, we were not able to obtain **K_Si_^W^[BOD_2_]** with the required purity. The organotin functionalization of POMs leads to the formation of robust hybrids since the lacuna of monovacant POMs is generally well-suited for the incorporation of one Sn^4+^ atom.^3^ We thus investigated the functionalization of the organotin polyoxotungstate and polyoxomolybdate platforms **K_Sn_^W^[I]** and **K_Sn_^Mo^[I]** with **BOD_2_-H**. In both cases stable hybrids of appropriate purity were obtained. The coupling reactions between the POM hybrid platforms bearing the iodoaryl moieties (*i.e.***K_Si_^W^[I]**, **K_Sn_^Mo^[I]** and **K_Sn_^W^[I]**) were performed following conditions that we previously established. Typically, Sonogashira C–C cross coupling occurs at 70 °C in *ca.* 30 min under microwave activation, in DMF containing triethylamine (20 equiv.), using [Pd(PPh)_3_Cl_2_] (8–10%) as catalyst source and Cu i (8–10%) as co-catalyst. An excess of the alkyne terminated-bodipy is required for the total conversion of the starting POM. For the synthesis of **K_Sn_^W^[BOD_2_]** and **K_Sn_^Mo^[BOD_2_]** a second loading of **BOD_2_-H** into the reaction mixture was performed after 15 min, due to the inevitable decomposition of **BOD_2_-H** under these conditions.

### Electrochemistry

**Fig. 1:**
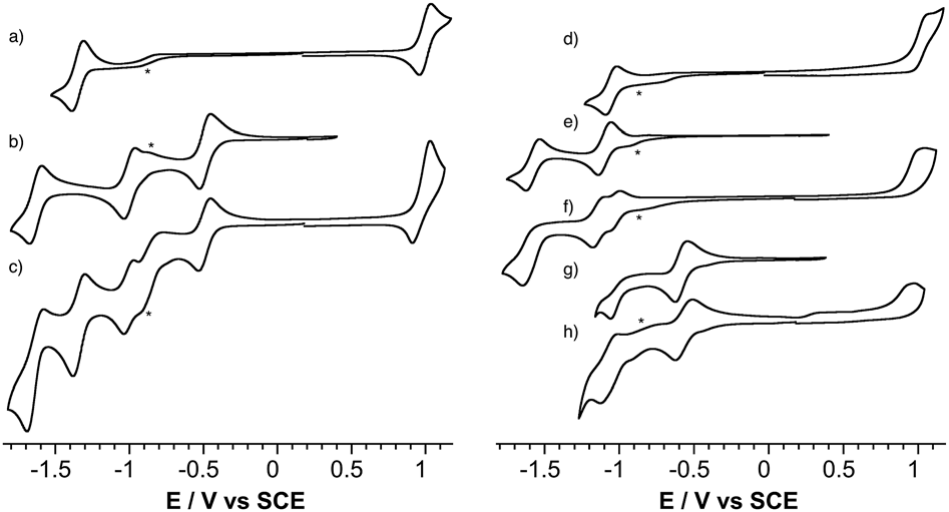
Cyclic voltammograms of *ca.* 0.5 mM solutions of reported compounds in DCM containing 0.1 M of TBAPF_6_: (a) **BOD_1_-TMS**, (b) **K_Si_^W^[I]**, (c) **K_Si_^W^[BOD_1_]**, (d) **BOD_2_-TMS** (e) **K_Sn_^W^[I]**, (f) **K_Sn_^W^[BOD_2_]**, (g) **K_Sn_^Mo^[I]**, (h) **K_Sn_^Mo^[BOD_2_]**. Scan rate, 20 mV s^–1^; working electrode, glassy carbon; reference electrode, SCE. The minor peaks noted with an asterisk are observed in the blank and correspond to residual traces of oxygen and/or solvent impurities.

The redox properties of the POM-bodipy dyads, their related POM hybrids and bodipy references were investigated by cyclic voltammetry and, in cases of irreversible redox processes, by differential pulse voltammetry in deoxygenated dichloromethane (DCM) with tetrabutylammonium hexafluorophosphate (TBAPF_6_) as the supporting electrolyte in a standard three-electrode cell, composed of a glassy carbon working electrode, a platinum counter electrode, and a saturated calomel reference electrode (SCE) (Fig. 1 and Table 1).

**Table 1:** Standard potentials (*vs.* SCE, in V)^*a*^ and peak-to-peak separation (mV) of the redox processes for the reported hybrids and reference compounds in DCM containing 0.1 M TBAPF_6_

Compound	[BOD]^+^/[BOD]	[BOD]/[BOD]^–^	POM/POM + 1 e	POM + 1 e/POM + 2 e
**BOD_1_-TMS**	1.00 (80)	–1.35 (80)		
**K_Si_^W^[I]**			–0.49 (70)	–1.00 (70)
**K_Si_^W^[BOD_1_]**	0.98	–1.34 (80)	–0.49 (80)	–1.00 (70)
**BOD_2_-TMS**	1.02	–1.05 (80)		
**K_Sn_^W^[I]**			–1.10 (80)	–1.58 (90)
**K_Sn_^W^[BOD_2_]**	1.02	–1.00	–1.11	–1.60
**K_Sn_^Mo^[I]**			–0.58 (80)	–1.03
**K_Sn_^Mo^[BOD_2_]**	0.98	–1.00	–0.57 (110)	–1.04

^*a*^The standard potentials of irreversible processes were determined by differential pulse voltammetry experiments (Fig. S7–S14).

First, it is noted that the redox potentials of the starting POM-based platforms are considerably shifted in DCM compared to acetonitrile,^3^ which outlines a very important effect of the organic solvent in the reduction processes of the polyanions. As previously mentioned when one compares the reduction potentials of the polyoxotungstates **K_Si_^W^[I]***versus***K_Sn_^W^[I]**, it can be seen that the latter is more difficult to reduce by more than half a volt due to a one-charge difference between both oxoclusters. **BOD_1_-TMS** and **BOD_2_-TMS** both feature reversible monoelectronic oxidation and reduction processes. While the oxidation potential for each are rather similar, reduction of **BOD_2_-TMS** occurs at a significantly more positive potential than reduction of **BOD_1_-TMS** (Δ*E*_red_ = 300 mV). Indeed, in **BOD_1_-TMS** the phenyl unit is twisted for steric reasons, which decouples it from the π system of the bodipy unit,^29^ while in **BOD_2_-TMS**, the π system of the bodipy unit extends over the alkynyl moieties, favouring its reduction. The redox properties of **K_Si_^W^[BOD_1_]** are very close to those of the reference compounds **K_Si_^W^[I]** and **BOD_1_-TMS**, as is often reported with our POM hybrid platforms.^3^,^19^,^23^,^26^,^30^ The shape of the oxidation waves of **K_Si_^W^[BOD_1_]** is characteristic of adsorption of the oxidized species at the working electrode. The redox properties of **K_Sn_^W^[BOD_2_]** and **K_Sn_^Mo^[BOD_2_]** are also similar to those of the reference compounds **K_Sn_^W^[I]**, **K_Sn_^Mo^[I]** and **BOD_2_-TMS**. As for **BOD_2_-TMS**, the oxidation of **K_Sn_^W^[BOD_2_]** and **K_Sn_^Mo^[BOD_2_]** are irreversible. In the reduction part, **K_Sn_^W^[BOD_2_]** displays two quasi-reversible processes followed by an irreversible process. The first one is attributed of the reduction of the bodipy unit while the two others correspond to monoelectronic reductions of the POM framework. In the reduction part, **K_Sn_^Mo^[BOD_2_]** displays two apparent quasi-reversible processes. The first one at –0.57 V *vs.* SCE is attributed to the reduction of the polyoxomolybdate framework, while the second at –1.04 V *vs.* SCE is attributed to quasi simultaneous reduction of the bodipy and the one-electron reduced POM (note that in differential pulse voltammetry experiment these two reduction processes are slightly separated with a maximum at –1.04 V and a shoulder at *ca.* –1.00 V *vs.* SCE attributed to the one-electron reduced POM and bodipy reduction respectively, Fig. S14†). The reduction potential comparison of **K_Sn_^Mo^[BOD_2_]***versus***K_Sn_^W^[BOD_2_]** thus suggests that the polyoxomolybdate core of **K_Sn_^Mo^[BOD_2_]** is more readily reduced than the polyoxotungstate core of **K_Sn_^W^[BOD_2_]** by the **BOD_2_** excited state.

### Electronic absorption and photophysical properties

**Fig. 2:**
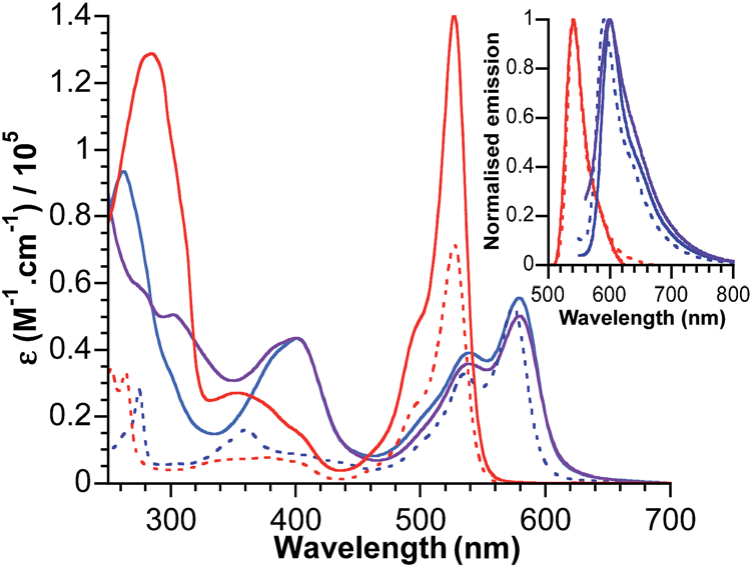
Absorbance and normalised emission (inset) of **BOD_1_-TMS** (dotted red), **BOD_2_-TMS** (dotted blue), **K_Si_^W^[BOD_1_]** (plain red), **K_Sn_^W^[BOD_2_]** (plain blue) and **K_Sn_^Mo^[BOD_2_]** (plain purple) in DCM.

The electronic absorption spectra for bodipy reference compounds and POM–bodipy hybrids dissolved in DCM are shown in Fig. 2. The absorption spectra of the hybrids are dominated by the bodipy absorption in the visible range, since the POM itself only absorbs in the UV part. The absorption profiles show a typical sharp bodipy-centred *S*_0_–*S*_1_ absorption with a maximum at 527 nm for **BOD_1_-TMS** and **K_Si_^W^[BOD_1_]**. For **BOD_2_-TMS**, **K_Sn_^W^[BOD_2_]** and **K_Sn_^Mo^[BOD_2_]**, the absorption profile is red-shifted due to an increase of the π system resulting from the presence of the alkynyl group at the *meso*-position. Note that the maximum bodipy absorption of **K_Sn_^W^[BOD_2_]** and **K_Sn_^Mo^[BOD_2_]** is slightly red-shifted (5 nm) compared to **BOD_2_-TMS** due to the participation of the aryl tin unit into the π system of the bodipy unit. A summary of the photophysical data is presented in Table 2.

**Table 2:** Photophysical data for bodipy references and POM–bodipy hybrids, recorded in DCM. *E*_0–0_ was calculated from the intercept between the absorption and emission spectra

	Absorption *λ*_max_ (nm)	Emission *λ*_max_ (nm)	*E*_0–0_ (eV)	*Φ*_FL_
**BOD_1_-TMS**	527	540	2.33	0.67
**BOD_2_-TMS**	574	590	2.14	0.52
**K_Si_^W^[BOD_1_]**	527	541	2.33	0.07
**K_Sn_^W^[BOD_2_]**	579	600	2.10	0.22
**K_Sn_^Mo^[BOD_2_]**	579	597	2.11	0.06

The fluorescence quantum yield for **K_Si_^W^[BOD_1_]** and **K_Sn_^Mo^[BOD_2_]** decrease by *ca.* 90% of that of **BOD_1_-TMS** and **BOD_2_-TMS**, respectively. This quenching of the bodipy fluorescence is consistent with electron transfer to the polyoxometalate. For **K_Sn_^W^[BOD_2_]**, the fluorescence quantum yield decreases by *ca.* 60% relative to **BOD_2_-TMS**, suggesting charge-transfer (if operating) from bodipy to POM is less efficient (note that the presence of the aryl tin unit that participates into the π system of the bodipy unit slightly modifies its electronic properties and may account for the decrease in quantum yield of **K_Sn_^W^[BOD_2_]**).

**Fig. 3:**
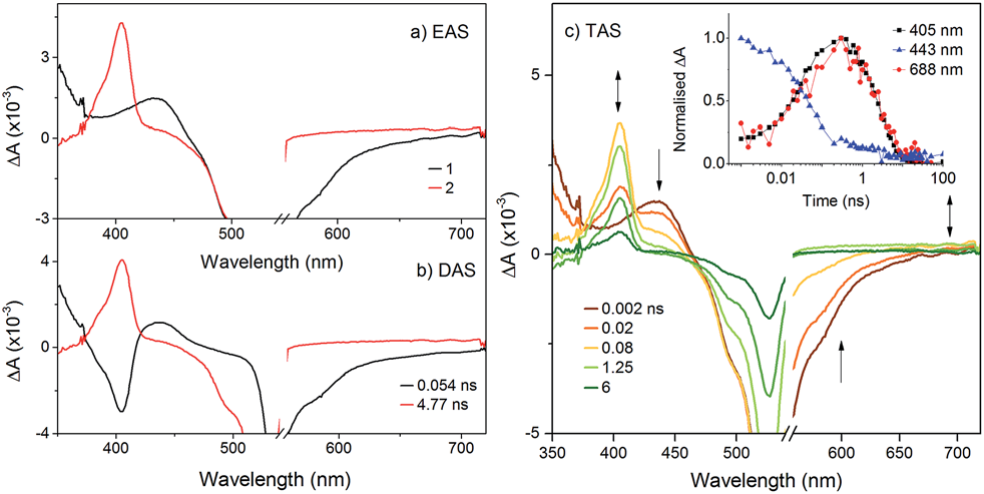
Transient absorption spectra of **K_Si_^W^[BOD_1_]** in DCM following excitation at 540 nm: (a) evolution associated difference spectra and; (b) decay associated difference spectra derived from global analysis; (c) transient absorption difference spectra at selected time delays after excitation, with single-point decays at 405 nm, 443 nm and 688 nm (inset).

To probe the fate of the bodipy excited state in these hybrids, transient optical and infrared absorption spectroscopy was performed on DCM solutions of the hybrids. The results are summarised in Table 3. To extract the intermediate, global analysis of the spectra was performed using the program OPTMUS, in which the transients at all detection wavelengths are analysed simultaneously with a single set of exponential functions.^31^ The transient absorption spectra of **K_Si_^W^[BOD_1_]** at various time delays after excitation at 540 nm are shown in Fig. 3. At short delay times, a bleach centered at 520 nm forms, consistent with the depletion of the bodipy ground state. The first transient species resembles the excited bodipy (spectra for **BOD_1_-TMS** and **BOD_2_-TMS** are provided in the ESI for comparison, Fig. S16 and 17†) and decays with *τ*_1_ = 54 ps, as a second transient species with a sharp absorption at 400 nm grows in over the same timescale and decays with *τ*_2_ = 4.8 ns. The absorption profile of this second transient is consistent with the oxidised bodipy generated by spectroelectrochemical methods (Fig. S15†). Furthermore, this transient also features a broad absorption starting at 600 nm and extending past 700 nm, which is characteristic of a reduced polyoxometalate, and is hence assigned to the charge-separated state, [BOD^+^-POM(+1 e^–^)].

**Table 3:** Driving force for charge-separation (Δ*G*_CS_) and recombination (Δ*G*_rec_) for POM–bodipy hybrids. Δ*G*_CS_ = *E*(BOD*/BOD^+^) – *E*(POM/POM^–^), Δ*G*_rec_ = *E*(POM/POM^–^) – *E*(BOD^+^/BOD). (BOD*/BOD^+^) = *E*(D/D^+^) – *E*_0–0_ (Note that the work terms for electrostatic interactions are neglected since they are estimated to be below 0.1 eV)

	–Δ*G*_CS_ (eV)	–Δ*G*_rec_ (eV)	*τ*_CS_ (ps)	*τ*_rec_ (ns)
**K_Si_^W^[BOD_1_]**	0.86	1.47	54	4.8
**K_Sn_^W^[BOD_2_]**	–0.03	2.13	—	—
**K_Sn_^Mo^[BOD_2_]**	0.56	1.55	180	0.52

**Fig. 4:**
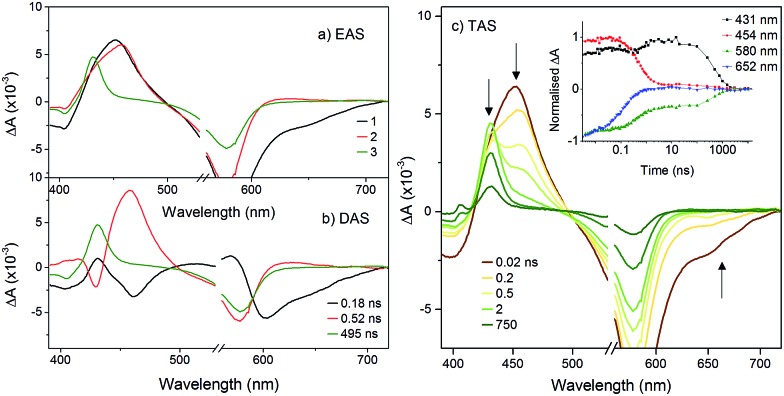
Transient absorption spectra of **K_Sn_^Mo^[BOD_2_]** in DCM following excitation at 540 nm: (a) evolution associated difference spectra and; (b) decay associated difference spectra derived from global analysis; (c) transient absorption difference spectra at selected time delays after excitation, with single-point decays at 431 nm, 454 nm, 580 nm and 652 nm (inset).

The transient spectra for **K_Sn_^Mo^[BOD_2_]** in DCM were more complex since a three component model was needed to accurately fit the relaxation dynamics of this hybrid. The first component (*τ*_1_ = 180 ps) in the evolution-associated difference spectra (EAS) has the same general shape as the **BOD_2_-TMS** excited state (Fig. S17†), and contains a negative signal from the stimulated emission. The second component (*τ*_2_ = 520 ps) is red-shifted (by 6 nm) relative to the first one and does not contain a contribution from the stimulated emission. We have assigned this to the charge-separated state, [BOD^+^-POM(+1 e^–^)] since the electron transfer from the **BOD_2_** to the polyoxomolybdate core of **K_Sn_^Mo^[BOD_2_]** is thermodynamically favourable and the absorption profile of this second transient is consistent with the oxidised bodipy (Fig. S15†) (Fig. 4).

This second species decays to form a long-lived component (*τ*_3_ = 495 ns), attributed to a bodipy triplet excited state. We have previously observed this behaviour for a triphenylamine–bodipy conjugate, arising from photoinduced charge-separation followed by recombination to the triplet state.^32^

**Fig. 5:**
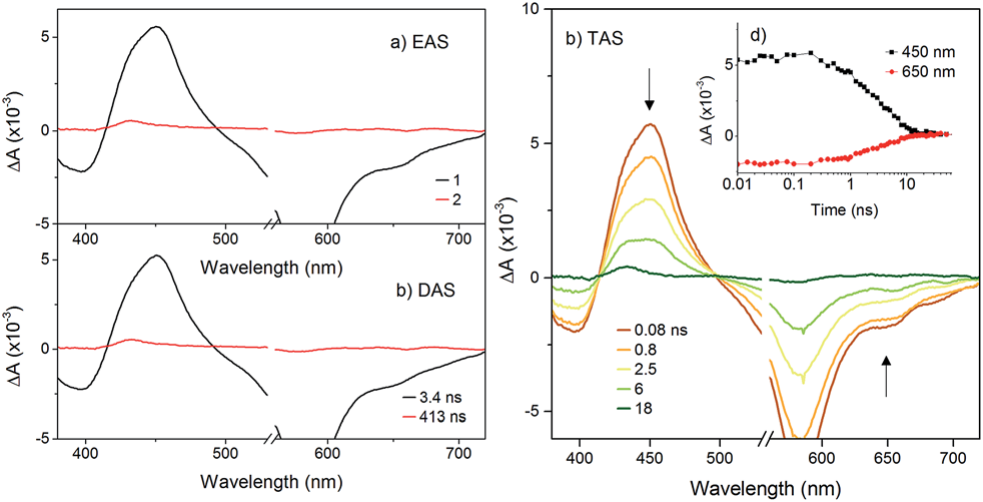
Transient absorption spectra of **K_Sn_^W^[BOD_2_]** in dichloromethane following excitation at 540 nm: (a) evolution associated difference spectra and; (b) decay associated difference spectra derived from global analysis; (c) transient absorption difference spectra at selected time delays after excitation, with single-point decays at 450 nm and 650 nm (inset).

The transient absorption spectra of **K_Sn_^W^[BOD_2_]** in DCM were much simpler (Fig. 5) since electron transfer from the **BOD_2_** to the polyoxotungstate core is thermodynamically not favourable. These transient spectra are initially similar in shape to those of the early transient spectra of **K_Sn_^Mo^[BOD_2_]**, in agreement with a bodipy centred excited state^25^ and decays with *τ*_1_ = 3.4 ns. A second minor transient signal at time delays exceeding 10 ns, which resembles that of the long-lived species observed with **K_Sn_^Mo^[BOD_2_]**, is observed (*τ*_2_ = 410 ns). This suggests a small concentration of the bodipy centered triplet state is formed, possibly caused by a heavy atom effect from the appended POM.

**Fig. 6:**
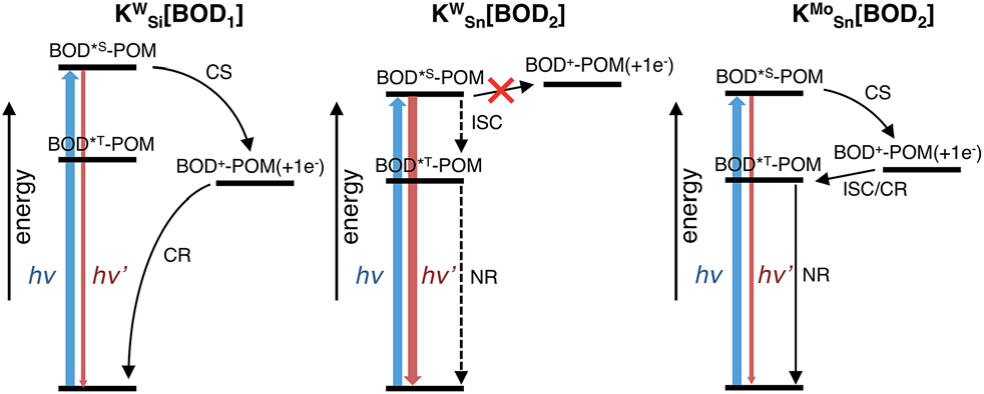
Energy level diagram of POM–bodipy hybrids in dichloromethane. CS: charge separation; CR: charge recombination; ICS: intersystem crossing; NR: non-radiative decay. The dotted arrows indicate poorly effective deactivation pathways. **K_Si_^W^[BOD_1_]**: *E*(BOD*^S^-POM) = 2.33 eV; *E*(BOD^+^-POM(+1 e^–^)) = 1.47 eV; *E*(BOD*^T^-POM) ∼1.65 eV. **K_Sn_^W^[BOD_2_]**: *E*(BOD*^S^-POM) = 2.10 eV; *E*(BOD^+^-POM(+1 e^–^)) = 2.13 eV; *E*(BOD*^T^-POM) ∼1.45 eV. **K_Sn_^Mo^[BOD_2_]**: *E*(BOD*^S^-POM) = 2.11 eV; *E*(BOD^+^-POM(+1 e^–^)) = 1.55 eV; *E*(BOD*^T^-POM) ∼1.45 eV.

To compare our results with those we obtained previously for all-organic bodipy systems,^32^,^33^ we also performed time-resolved infrared spectroscopy (Fig. S22†). The dynamics agree with those recorded in the visible region. In the spectra for **K_Sn_^Mo^[BOD_2_]** a sharp, long lived band at 1530 cm^–1^ is present. The shape and the lifetime of this band are in agreement with our previous work and supports our assignment of the long-lived species as the bodipy triplet excited state.^33^ The fact that the charge-separated state recombines *via* a bodipy-centered triplet state in **K_Sn_^Mo^[BOD_2_]** but directly to the ground state in **K_Si_^W^[BOD_1_]** is probably due to the slightly higher energy of the CS state (Fig. 6) and the lower energy of the bodipy triplet state in **K_Sn_^Mo^[BOD_2_]** compared to **K_Si_^W^[BOD_1_]**. Phosphorescence from bodipy is rarely observed, but Harriman *et al.* recorded *λ*_phos_ = 750 nm (1.65 eV) in a molecular dyad system incorporating a structurally similar bodipy to **BOD_1_** appended to zinc terpyridine.^34^ Assuming that the triplet energy is similar, this places it 150–200 meV higher than the charge-separated state in **K_Si_^W^[BOD_1_]**.

As the POM and the bodipy are considerably electronically decoupled, it is possible to separate processes centered on each component of the POM–bodipy hybrids and determine the driving forces for charge-separation and recombination, as presented in Table 3.

For **K_Si_^W^[BOD_1_]** and **K_Sn_^Mo^[BOD_2_]** there is a large driving force for charge-separation (–Δ*G*_CS_, 0.86 and 0.56 eV respectively), which occurs with a significantly faster rate for **K_Si_^W^[BOD_1_]** than **K_Sn_^Mo^[BOD_2_]**. This is consistent with charge separation in the Marcus normal region (as described for the POM–iridium hybrids).^23^ In **K_Si_^W^[BOD_1_]**, the organic spacer between the POM and the bodipy introduces an additional phenyl ring compared to **K_Sn_^Mo^[BOD_2_]** increasing thus the POM-bodipy distance of *ca.* 4 Å. Furthermore, in **K_Si_^W^[BOD_1_]**, the organic spacer is electronically decoupled from the π system of the bodipy unit and thus acts as an insulator.

By contrast, in **K_Sn_^Mo^[BOD_2_]** the π system of the bodipy unit extends over the aryl tin ring *i.e.* at the vicinity of the polyoxomolybdate core. According to Marcus theory, the rate constant for an electron transfer process of a supramolecular system in the non-adiabatic limit (*i.e.* when its different elements are poorly electronically coupled) can be expressed by the following equation:^35^,^36^ 
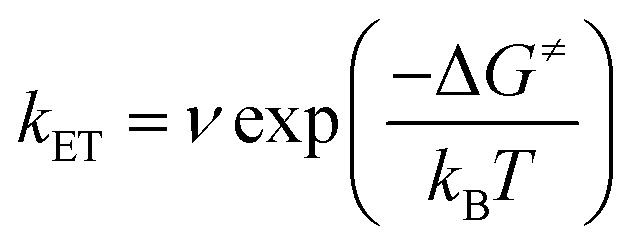
where *ν* is an electronic factor that is proportional to the overlap between the electronic wavefunctions of the donor and acceptor units. This electronic factor should be considerably more important for **K_Sn_^Mo^[BOD_2_]** than for **K_Si_^W^[BOD_1_]**. The fact that photoinduced charge separation is significantly faster in **K_Si_^W^[BOD_1_]** than in **K_Sn_^Mo^[BOD_2_]**, despite a lower electronic factor term, outlines the importance of the driving force (imposed by the redox potentials of the POM and the bodipy) on the photo-induced electron transfer in these molecular systems. In other words, a short organic spacer is not mandatory for efficient charge injection if the energy levels are well positioned. The photophysics (*i.e.* rapid formation and long-lifetime of the charge-separated state) of the photosensitized POM-based hybrids described here, particularly **K_Si_^W^[BOD_1_]**, are promising for applications in photoelectrochemical solar cells. Dye-sensitized NiO-based photocathodes are limited by the fast (<ns) rate of charge recombination at the oxide–dye interface.^37^,^38^ The tune-ability of the electronic properties of the photosensitizer and POM subunits to control the charge-transfer dynamics is, therefore, extremely attractive for implementing photosensitized POM-based hybrids in molecular photocathodes.

## Conclusions

New POM–bodipy conjugates were synthesized through post-functionalization of organosilyl and organotin POM derivatives. An advantage of these systems over *e.g.* dye-sensitized TiO_2_, is that the electronic properties of both the donor and acceptor can be specifically tuned. In these photoactive hybrids the redox properties of the POM, the bodipy and the spacer length were modified in order to evaluate the effect of these parameters on the kinetics of photoinduced electron transfer. The transient absorption spectroscopy unequivocally indicates the occurrence of photoinduced electron transfer from the bodipy to the POM for hybrids displaying the best electron accepting properties, with kinetics up to *ca.* 2 × 10^10^ s^–1^, constituting the first example of charge-separated state on noble metal-free covalent POM–PS conjugates. While POMs are drawing a growing attention in the field of artificial photosynthesis and molecular electronics, fundamental insights on their kinetics of intramolecular electron injection into the POM unit within POM–PS conjugates are scare.^18^,^23^,^39^,^40^ For instance the effects of the solvent and their associated counter-ions on their reorganization energy remain unexplored, the only experimental studies being yet limited to outer-sphere chemical and electrochemical reduction of POMs.^41^,^42^ The present system should allow filling this shortfall owing to their modular design. The long lifetime of the charge separated state is also an exciting prospect for integrating these systems into photocathodes, since electron-hopping between POM units or charge-transfer to a substrate or catalyst would be competitive with recombination.

## Conflicts of interest

There are no conflicts to declare.

## Supplementary Material

Supplementary information
