# Supplementary material for: Rapid photoinduced charge injection into covalent polyoxometalate–bodipy conjugates
Source: Chem Sci. 2018 Jun 1;9(25):5578–84. doi: 10.1039/c8sc00862k (PMC6048759; doi:10.1039/c8sc00862k)
Supplement: Supplementary file 1 [file SC-009-C8SC00862K-s001.pdf]

Electronic Supplementary Material (ESI) for Chemical Science.
This journal is © The Royal Society of Chemistry 2018

Rapid photoinduced charge injection into covalent polyoxometalate-bodipy conjugates

Fiona A. Black, Aurélie Jacquart, Georgios Toupalas, Sandra Alves, Anna Proust, Ian P. Clark, Elizabeth A. Gibson,* Guillaume Izzet*

Contents

1. **General methods**

2. **Synthesis and characterizations of K^{W}_{Si}[BOD$_1$]**

Figure S1. ^{1}H NMR (300 MHz) ^{31}P and ^{19}F NMR (121 and 282 MHz respectively, framed inset) spectra of **K^{W}_{Si}[BOD$_1$]** in CD_3CN.
Figure S2. Comparison of experimental (upper trace) and calculated (lower trace) isotopic peaks for the $[PW_{11}O_{39}\{O(SiC_{31}H_{30}N_2BF_2)_2\}]^{3-}$ ion of **K^{W}_{Si}[BOD$_1$]**.

3. **Synthesis and characterizations of K^{W}_{Sn}[BOD$_2$]**

Figure S3. ^{1}H NMR (300 MHz) ^{31}P and ^{19}F NMR (121 and 282 MHz respectively, framed inset) spectra of **K^{W}_{Sn}[BOD$_2$]** in CD_3CN.
Figure S4. Comparison of experimental (upper trace) and calculated (lower trace) isotopic peaks for the ions $[PW_{11}O_{39}SnC_{25}H_{26}BF_2N_2]^{4-}$ (left) and $[PW_{11}O_{39}SnC_{25}H_{26}BF_2N_2.TBA]^{3-}$ (right) of **K^{W}_{Sn}[BOD$_2$]**.

4. **Synthesis and characterizations of K^{Mo}_{Sn}[BOD$_2$]**

Figure S5. ^{1}H NMR (300 MHz) ^{31}P and ^{19}F NMR (121 and 282 MHz respectively, framed inset) spectra of **K^{Mo}_{Sn}[BOD$_2$]** in CD_3CN.
Figure S6. Comparison of experimental (upper trace) and calculated (lower trace) isotopic peaks for the ion $[PMo_{11}O_{39}SnC_{25}H_{26}BF_2N_2]^{3-}$ of **K^{Mo}_{Sn}[BOD$_2$]**.

5. **Differential pulse voltammetry**

Figure S7. Differential pulse voltammograms of **BOD$_1$-TMS** in DCM containing 0.1 M $TBAPF_6$.
Figure S8. Differential pulse voltammograms of **K^{W}_{Si}[I]** in DCM containing 0.1 M $TBAPF_6$.
Figure S9. Differential pulse voltammograms of **K^{W}_{Si}[BOD$_2$]** in DCM containing 0.1 M $TBAPF_6$.
Figure S10. Differential pulse voltammograms of **BOD$_2$-TMS** in DCM containing 0.1 M $TBAPF_6$.
Figure S11. Differential pulse voltammograms of **K^{W}_{Sn}[I]** in DCM containing 0.1 M $TBAPF_6$.
Figure S12. Differential pulse voltammograms of **K^{W}_{Sn}[BOD$_2$]** in DCM containing 0.1 M $TBAPF_6$.
Figure S13. Differential pulse voltammograms of **K^{Mo}_{Sn}[I]** in DCM containing 0.1 M $TBAPF_6$.

Figure S14. Differential pulse voltammograms of $\mathbf{K^{Mo}{}_{Sn}[BOD_2]}$ in DCM containing 0.1 M $TBAPF_6$.

6. Spectroelectrochemistry

Figure S15. Absorption difference spectra of $\mathbf{BOD_1}$**-TMS**, $\mathbf{BOD_2}$**-TMS**, $\mathbf{K^{W}{}_{Si}[I]}$, $\mathbf{K^{W}{}_{Sn}[I]}$ and $\mathbf{K^{Mo}{}_{Sn}[I]}$ in 0.2 M $TBAPF_6$ DCM solution showing the evolution of the oxidised bodipys and reduced POMs generated electrochemically.

7. Transient absorption spectroscopy

Figure S16. Ultrafast transient absorption spectra of $\mathbf{BOD_1}$**-TMS** (red) and $\mathbf{BOD_2}$**-TMS** (black) in DCM solution, 50 ps after excitation at 540 nm.

Figure S17. Decay associated difference spectra (DAS) of $\mathbf{BOD_1}$**-TMS** (a) and $\mathbf{BOD_2}$**-TMS** (b), the evolution associated difference spectra (EAS) of $\mathbf{BOD_2}$**-TMS** (c) in DCM solution.

Figure S18. The DAS (a) and EAS (b) of $\mathbf{K^{W}{}_{Si}[BOD_1]}$ in DCM.

Figure S19. The DAS (a) and EAS (b) of $\mathbf{K^{W}{}_{Sn}[BOD_2]}$ in DCM.

Figure S20. The DAS (a) and EAS (b) of $\mathbf{K^{Mo}{}_{Sn}[BOD_2]}$ in DCM.

Figure S21. Comparison of experimental data with model extracted from the global analysis.

Figure S22. TRIR of POM-bodipy hydrids in DCM. Top: $\mathbf{K^{W}{}_{Si}[BOD_1]}$, middle: $\mathbf{K^{W}{}_{Sn}[BOD_2]}$, bottom: $\mathbf{K^{Mo}{}_{Sn}[BOD_2]}$.

8. Energy level diagram

Figure S24. Energy level diagram of POM-bodipy hybrids in dichloromethane.

9. References

General methods. Triethylamine (TEA) was dried over CaH_2 and freshly distilled under argon before use. Dimethylformamide (DMF) was purchased stored under argon over molecular sieve. Other solvents and reagents were obtained from commercial sources and used as received. Reactions, unless otherwise mentioned, were carried out under dry argon by using standard Schlenk-tube techniques. **[BOD$_1$-H]**,[1] **[BOD$_2$-H]**,[2] **K^W_{Si}[I]**,[3] **K^W_{Sn}[I]**,[4] **K^{Mo}_{Sn}[I]**[5] and [$Pd(PPh_3)_2Cl_2$][6] were synthesized according to literature procedures. Microwave assisted syntheses were performed at an ambient pressured reactor (Milestone Start S) equipped with a temperature control unit. The ^1H (300.3 MHz), {^1H} ^{31}P (121.5 MHz) and ^{19}F (282.4 MHz) NMR spectra were obtained at room temperature in 5 mm o.d. tubes on a Bruker AvanceII 300 spectrometer equipped with a QNP probehead. IR spectra were recorded from KBr pellets on a Jasco FT/IR 4100 spectrometer. Electrochemical studies were performed on an Autolab PGSTAT 100 workstation. A standard three electrode cell was used, which consisted of a working vitrous carbon electrode, an auxiliary platinum electrode and an aqueous saturated calomel electrode (SCE) equipped with a double junction. Spectroelectrochemical studies were conducted with the aid of an optically transparent thin-layer electrochemical cell (OTTLE). The OTTLE consisted of a platinum-mesh working electrode, a platinum-wire counter electrode, and a silver wire quasi-reference electrode. The ESI mass spectra were recorded using an LTQ Orbitrap hybrid mass spectrometer (Thermofisher Scientific, Bremen, Germany) equipped with an external ESI source operated in the negative ion mode. Spray conditions included a spray voltage of 3 kV, a capillary temperature maintained at 280 °C, a capillary voltage of −30 V, and a tube lens offset of −90 V. Sample solutions in acetonitrile (10 pmol.μL^{-1}) were infused into the ESI source by using a syringe pump at a flow rate of 180 $\mu L.h^{-1}$. Mass spectra were acquired in the Orbitrap analyzer with a theoretical mass resolving power (R_p) of 100 000 at m/z 400, after ion accumulation to a target value of 10^5 and a m/z range from m/z 300 to 2000. All data were acquired using external calibration with a mixture of caffeine, MRFA peptide and Ultramark 1600 dissolved in Milli-Q water/ HPLC grade acetonitrile (50/50, v/v). Elemental analyses were performed at the Institut de Chimie des Substances Naturelles, Gif sur Yvette, France. UV-visible absorption spectra were recorded on a Jasco V-670 equipped with a ETC-717 Peltier module. Photoluminescence spectroscopy measurements in solution were recorded using a Shimadzu RF-6000 spectro fluorophotometer. All measurements were performed using a quartz cuvette with a path length of 1 cm.

Ultrafast transient absorption spectroscopy. Time-resolved spectroscopy data were recorded using time-resolved multiple probe spectroscopy (TR^MPS), a brief description is provided below and full details are described in ref 7. Briefly, two Ti:sapphire amplifiers with repetition rates of 10 kHz and 1 kHz were synchronized using a common 65 MHz oscillator. The 1 kHz output was used as a pump and the 10 kHz as probe, to permit a pump−probe−probe−probe... data-recording scheme. The pump laser was tuned to 540 nm by optical parametric amplification (OPA). For transient absorption spectroscopy in the visible region, the probe pulse was provided by a white light continuum (WLC), which was generated by focusing 800 nm into CaF_2. For time-resolved infrared spectroscopy, the mid-IR probe was generated using an OPA with difference frequency mixing. The pump−probe time delay was controlled up to 100 µs using a combination of electronic and optical delay. Spot sizes in the sample region were ca. 150 and 50 µm for the pump and probe, respectively, with a pump energy of 20 nJ. For all measurements the pump polarization was set to magic angle relative to the probe. All spectra were recorded in solution cells (Harrick Scientific Products Inc.) with CaF_2 windows using a PTFE spacer (490 µm path length). These were filled with solutions of the hybrids in dichloromethane and the concentrations adjusted to give optical densities of *ca.* 0.3 at the excitation wavelength (490 µm path length). In all experiments the

cell was rastered in the two dimensions orthogonal to the direction of beam propagation to minimize localized sample decomposition. Molecular signals were verified to exhibit linear behaviour with respect to the pump intensity, indicating the kinetics reported correspond to single-photon absorption events. Four pump-probe-probe-probe cycles were averaged and the resulting data is displayed. Global analysis was performed using the program OPTIMUS[8]. The chirp correction was applied prior to global analysis. In global lifetime analysis, a model function for each detection wavelength is constructed. The optimisation is initated with some starting values used to calculate each of the basic functions. The amplitudes of each function is found by linear fitting using the Moore-Penrose pseudoinverse (MATLAB *pinv* function). The fit parameters are optimised iteratively to minimise the square of the residual norm. The amplitudes are plotted against the detection wavelength to yield the decay associated spectra.

Synthesis of K^{W}_{Si}[BOD$_1$]

A mixture of **K^{W}_{Si}[I]** (150 mg, 0.039 mmol), **BOD$_1$-H** (62 mg, 0.15 mmol, 4 equiv), CuI (0.73 mg, 3.8×10^{-3} mmol, 0.1 equiv) and [$PdCl_2(PPh_3)_2$] (2.7 mg, 3.9×10^{-3} mmol, 0.1 equiv) in 5 mL of dry DMF was prepared in a Schlenk tube under argon atmosphere. After careful degassing with argon for 10 minutes, freshly distilled TEA (80 mg, 0.79 mmol, 20 equiv) was added. The mixture was stirred at 70°C for 30 min, under microwave irradiation. The resulting dark red solution was precipitated with diethyl ether. The precipitate was filtered and redissolved in acetone (10 mL), and stirred with 5 mL of Amberlite IR-120 in the TBA^+ form. The resulting colored solution was filtered, concentrated to ca. 4 mL and added to 8 mL ethyl acetate. A slight precipitate was removed from the colored supernatant that precipitates by the addition of a large excess of diethyl ether, yielding a bright red powder (150 mg, Yield: 88%). ^{1}H NMR (CD_3CN): δ (ppm) 7.90 (d, J = 8.3 Hz, 4H), 7.73 (d, J = 8.3 Hz, 4H), 7.68 (d, J = 8.3 Hz, 4H), 7.39 (d, J = 8.3 Hz, 4H), 3.09 (m, 24H), 2.48 (s, 12H), 2.34 (q, J = 7.6 Hz, 8H), 1.62 (m, 24H), 1.37 (s, 12H), 1.36 (h, J = 7.3 Hz, 24H), 0.98 (t, J = 7.3 Hz, 36H), 0.98 (t, J = 7.6 Hz, 12H). ^{31}P (CD_3CN): δ (ppm) -12.08 (s). ^{19}F (CD_3CN): δ (ppm) -145.04 (q, J_{BF} = 32.7 Hz). IR (KBr, cm^{-1}): ν 2963 (s), 2932 (m), 2873 (m), 2217 (vw), 1626 (w), 1597 (w), 1541 (s), 1476 (s), 1405 (m), 1386 (m), 1321 (m), 1274 (w), 1195 (s), 1161 (w), 1147 (w), 1111 (s), 1066 (s), 1039 (s), 1017 (w), 965 (vs), 873 (vs), 825 (vs), 765 (m), 711 (m), 644 (m), 600 (m), 523 (m), 388 (s). MS (ESI-): m/z POM=$PW_{11}Si_2O_{40}C_{62}H_{60}N_4B_2F_4$ $[POM]^{3-}$ 1235.89, calcd 1235.89. Elemental analysis for $PSi_2W_{11}O_{40}C_{110}H_{168}N_7B_2F_4$ (%): calcd C 29.79; H 3.82; N 2.21; found C 30.06; H 3.62; N 2.22.

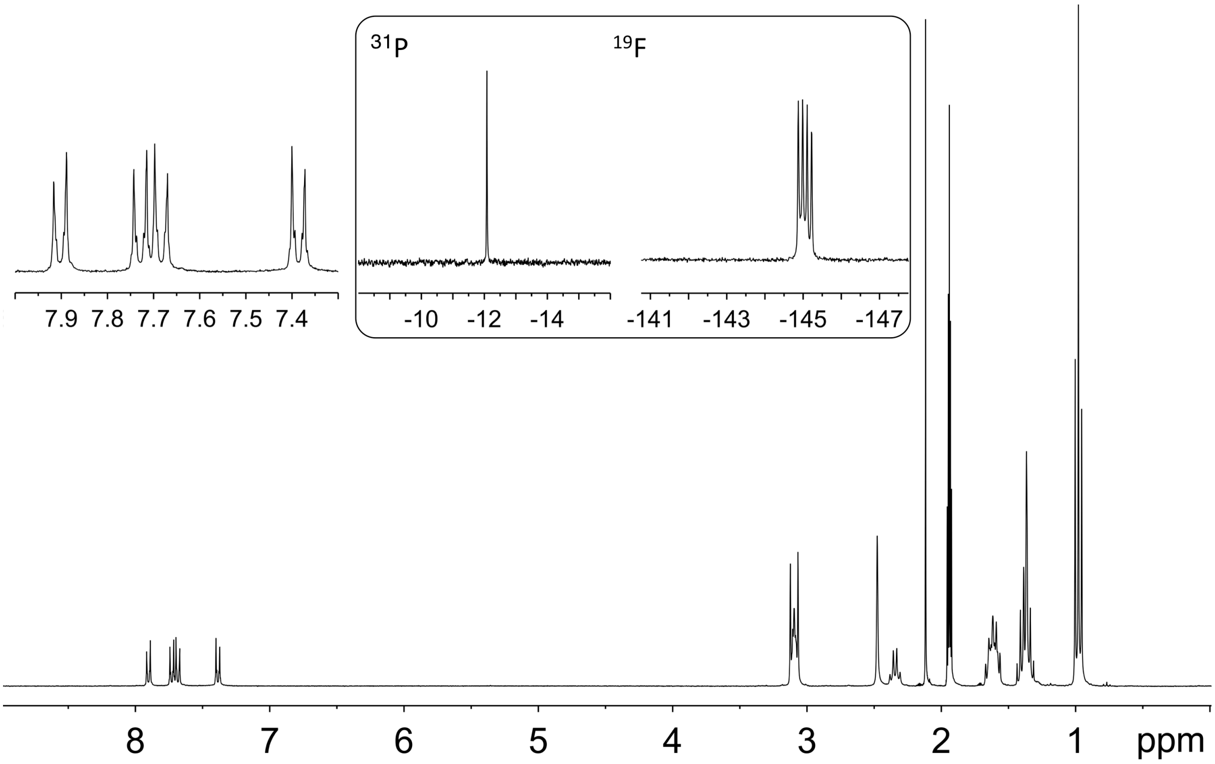

Figure S1. ^{1}H NMR (300 MHz) ^{31}P and ^{19}F NMR (121 and 282 MHz respectively, framed inset) spectra of $\mathbf{K^{W}_{Si}[BOD_1]}$ in CD_3CN.

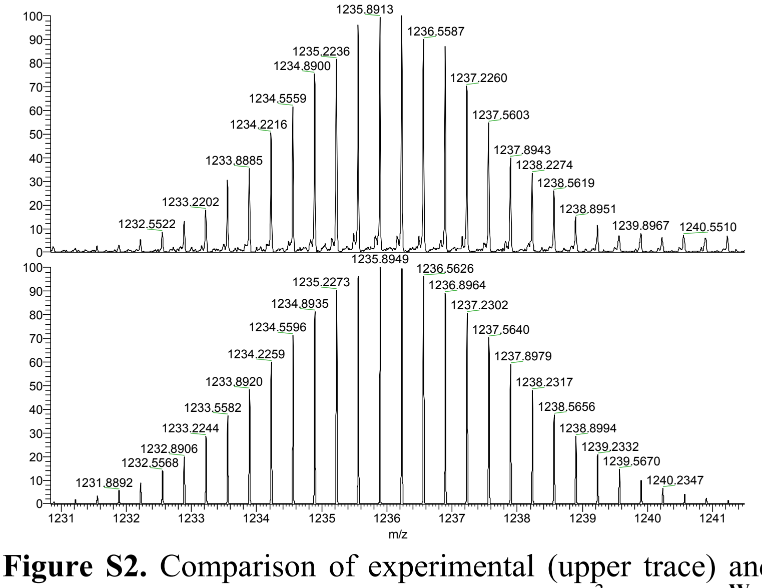

Figure S2. Comparison of experimental (upper trace) and calculated (lower trace) isotopic peaks for the $[PW_{11}O_{39}\{O(SiC_{31}H_{30}N_2BF_2)_2\}]^{3-}$ ion of $\mathbf{K^{W}_{Si}[BOD_1]}$.

Synthesis of K^{W}_{Sn}[BOD$_2$]

A mixture of **K^{W}_{Sn}[I]** (150 mg, 0.039 mmol), **BOD$_2$-H** (26 mg, 0.079 mmol, 2 equiv), CuI (0.60 mg, 3.2×10^{-3} mmol, 0.08 equiv) and [PdCl$_2$(PPh$_3$)$_2$] (2.3 mg, 3.2×10^{-3} mmol, 0.08 equiv) in 5 mL of dry DMF was prepared in a Schlenk tube under argon atmosphere. After careful degassing with argon for 10 minutes, freshly distilled TEA (80 mg, 0.78 mmol, 20 equiv) was added. The mixture was stirred at 70°C for 15 min, under microwave irradiation. A second loading of **BOD$_2$-H** (13 mg, 0.040 mmol, 1 equiv) was then added under argon and the mixture was heated at 70°C for additional 15 min. Tetrabutylammonium bromide (0.50 g, 1.55 mmol, 40 equiv) was added. The product was then precipitated by the addition of an excess of diethyl ether. After centrifugation, the solid was dissolved in a minimum of acetonitrile and precipitated with an excess of ethanol. The solid was redissolved in acetonitrile (4 mL), filtered and added to 2 mL of AcOEt. A precipitate was separated from the surpernatant that was precipitated by addition of an excess of diethyl ether to yield a dark purple solid (90 mg, Yield: 55%). A slight excess of TBA is observed in the ^{1}H NMR spectrum and confirmed by elemental analysis. ^{1}H NMR (CD$_3$CN): δ (ppm) 7.81 (d+dd, J_{HH} = 8.1 Hz, J_{SnH} = 96 Hz, 2H), 7.75 (d+dd, J_{HH} = 8.1 Hz, J_{SnH} = 42 Hz, 2H), 3.13 (m, 36H), 2.55 (s, 6H), 2.47 (s, 6H), 2.46 (q, J = 7.5 Hz, 4H), 1.63 (m, 36H), 1.39 (h, J = 7.3 Hz, 36H), 1.06 (t, J = 7.5 Hz, 6H), 0.98 (t, J = 7.3 Hz, 54H). ^{31}P (CD$_3$CN): δ (ppm) -10.95 (s+d, J_{SnP} = 24.4 Hz). ^{19}F (CD$_3$CN): δ (ppm) -144.77 (q, J_{BF} = 32.6 Hz). IR (KBr, cm^{-1}): ν 2961 (s), 2932 (m), 2872 (m), 2206 (w), 1633 (w), 1540 (s), 1472 (s), 1383 (m), 1321 (m), 1264 (w), 1195 (s), 1114 (w), 1070 (s), 963 (vs), 886 (s), 813 (vs), 801 (vs), 717 (w), 705 (w), 661 (w), 515 (w), 381 (s). MS (ESI-): m/z POM=$PW_{11}O_{39}SnC_{25}H_{26}BF_2N_2$ [POM]$^{4-}$ 799.588 calcd 799.900; [POM]$^{3-}$ 1066.451, calcd 1066.453; [POM.TBA]$^{3-}$ 1147.213, calcd 1147.215. Elemental analysis for $PW_{11}O_{39}C_{89}H_{170}N_6BF_2$.(TBABr)$_{0.5}$ (%): calcd C 26.91; H 4.38; N 2.10; found 27.08; H 4.11; N 2.23.

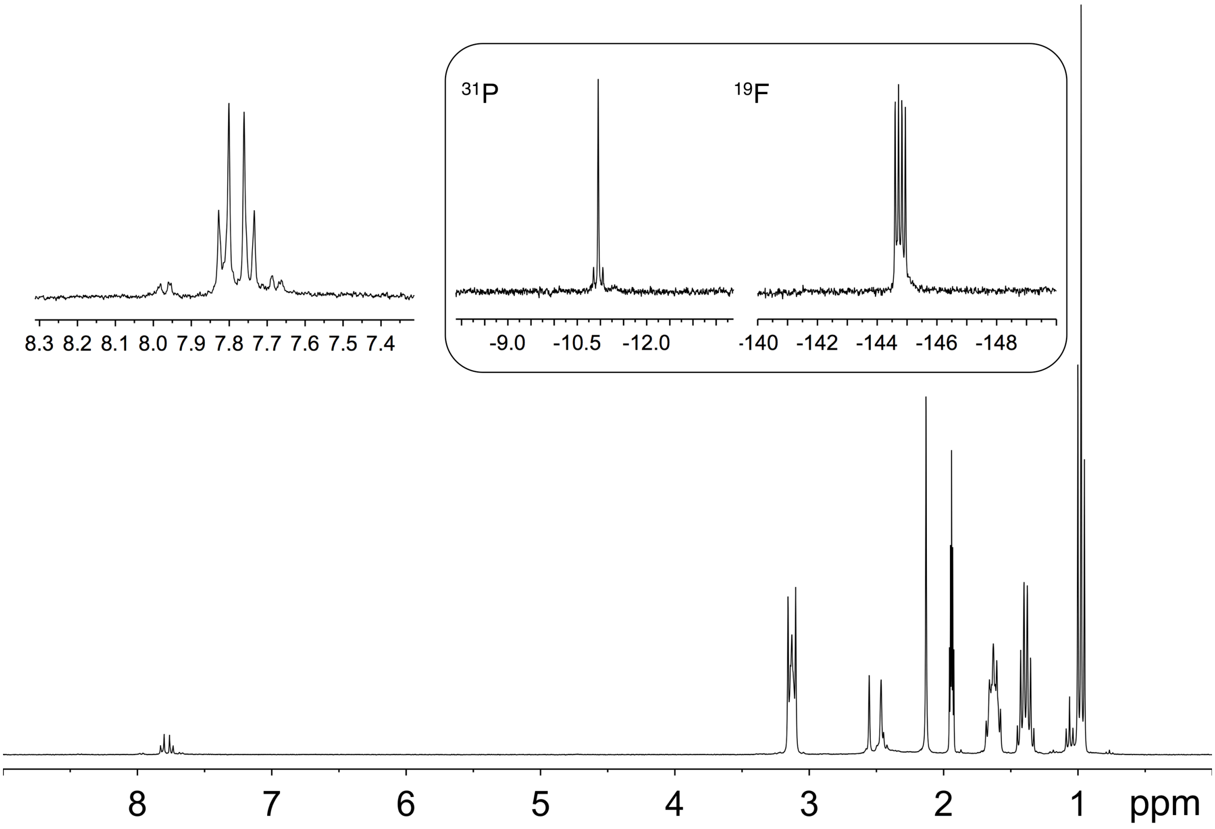

Figure S3. ^{1}H NMR (300 MHz) ^{31}P and ^{19}F NMR (121 and 282 MHz respectively, framed inset) spectra of $\mathbf{K^{W}_{Sn}[BOD_2]}$ in CD_3CN.

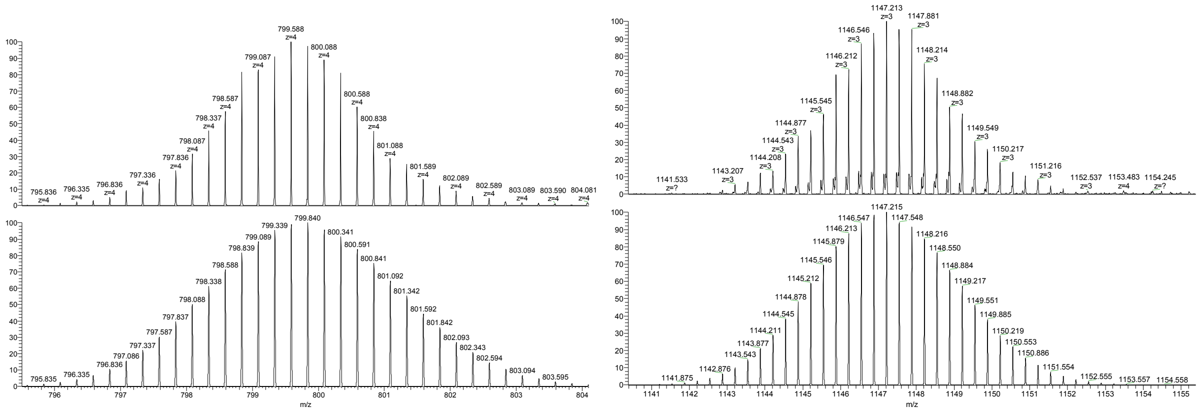

Figure S4. Comparison of experimental (upper trace) and calculated (lower trace) isotopic peaks for the ions $[PW_{11}O_{39}SnC_{25}H_{26}BF_2N_2]^{4-}$ (left) and $[PW_{11}O_{39}SnC_{25}H_{26}BF_2N_2.TBA]^{3-}$ (right) of $\mathbf{K^{W}_{Sn}[BOD_2]}$.

Synthesis of $K^{Mo}{}_{Sn}[BOD_2]$

A mixture of **$K^{Mo}{}_{Sn}$[I]** (100 mg, 0.033 mmol), **BOD_2-H** (22 mg, 0.067 mmol, 2 equiv), CuI (0.50 mg, 2.6×10^{-3} mmol, 0.08 equiv) and $[PdCl_2(PPh_3)_2]$ (1.9 mg, 2.7×10^{-3} mmol, 0.08 equiv) in 3 mL of dry DMF was prepared in a Schlenk tube under argon atmosphere. After careful degassing with argon for 10 minutes, freshly distilled TEA (67 mg, 0.66 mmol, 20 equiv) was added. The mixture was stirred at 70°C for 15 min, under microwave irradiation. A second loading of **BOD_2-H** (11 mg, 0.034 mmol, 1 equiv) was then added under argon and the mixture was heated at 70°C for additional 15 min. Tetrabutylammonium bromide (0.50 g, 1.55 mmol, 47 equiv) was added. The product was then precipitated by the addition of an excess of diethyl ether. After centrifugation, the solid was dissolved in a minimum of acetonitrile and precipitated with an excess of ethanol. The solid was redissolved in acetonitrile (4 mL), filtered and added to 2 mL of AcOEt. A precipitate was separated from the surpernatant that was precipitated by addition of an excess of diethyl ether to yield a dark purple solid (68 mg, Yield: 65%). As for **$K^{W}{}_{Sn}[BOD_2]$** a slight excess of TBA is present in the sample. ^{1}H NMR (CD_3CN): δ (ppm) 7.77 (d+dd, J_{HH} = 8.1 Hz, J_{SnH} = 93 Hz, 2H), 7.70 (d+dd, J_{HH} = 8.1 Hz, J_{SnH} = 43 Hz, 2H), 3.13 (m, 34H), 2.55 (s, 6H), 2.47 (s, 6H), 2.46 (q, J = 7.6 Hz, 4H), 1.64 (m, 34H), 1.39 (h, J = 7.3 Hz, 34H), 1.06 (t, J = 7.6 Hz, 6H), 0.98 (t, J = 7.3 Hz, 51H). ^{31}P (CD_3CN): δ (ppm) -2.44 (s+d, J_{SnP} = 36.6 Hz). ^{19}F (CD_3CN): δ (ppm) -144.72 (q, J_{BF} = 32.5 Hz). IR (KBr, cm^{-1}): ν 2961 (s), 2931 (m), 2872 (m), 2204 (w), 1634 (w), 1540 (s), 1473 (s), 1384 (m), 1321 (m), 1265 (w), 1196 (s), 1113 (w), 1062 (s), 1035 (m), 974 (w), 944 (vs), 867 (s), 807 (vs), 789 (vs), 534 (w), 385. MS (ESI-): m/z POM=$PMo_{11}O_{39}SnC_{25}H_{26}BF_2N_2$ $[POM]^{3-}$ 743.952, calcd 743.953. Elemental analysis for $PMo_{11}O_{39}C_{89}H_{170}N_6BF_2.(TBABr)_{0.25}$ (%): calcd C 34.03; H 5.50; N 2.67; found 34.31; H 5.26; N 2.80.

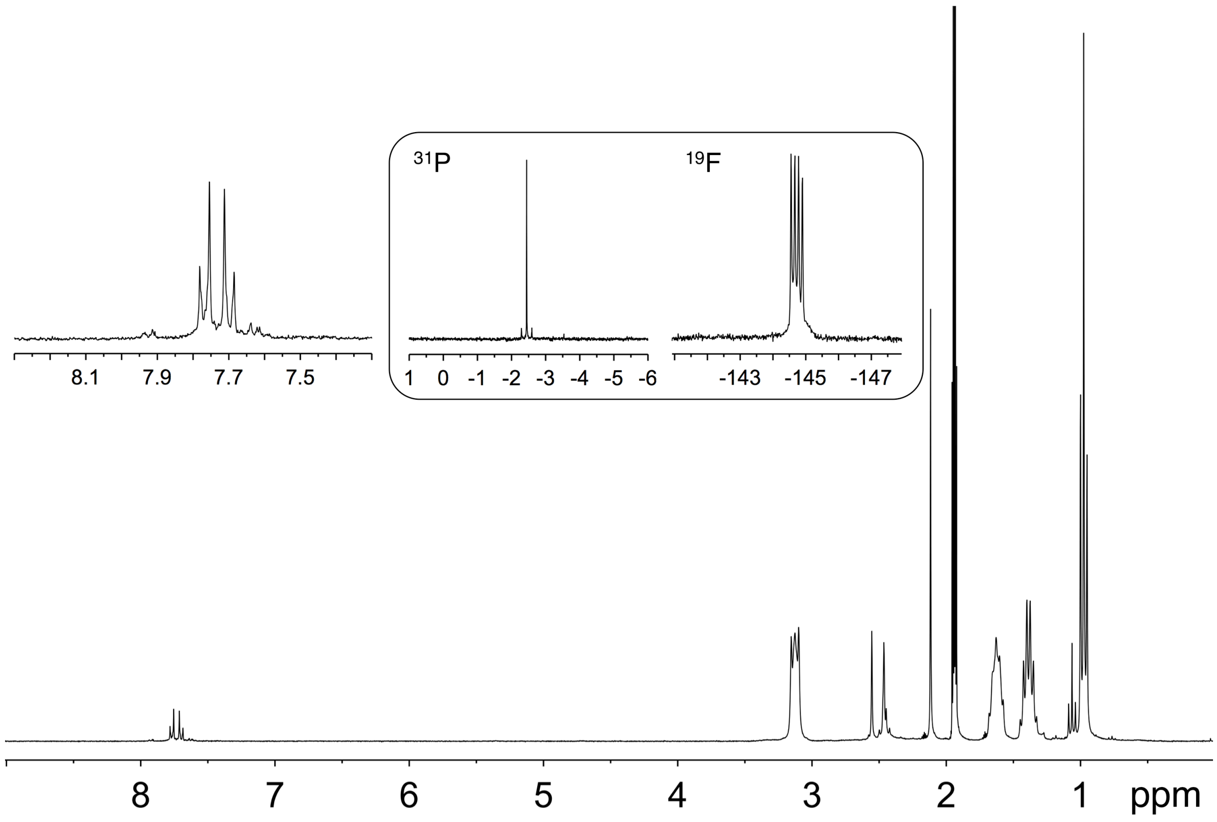

Figure S5. ^{1}H NMR (300 MHz) ^{31}P and ^{19}F NMR (121 and 282 MHz respectively, framed inset) spectra of **$K^{Mo}{}_{Sn}[BOD_2]$** in CD_3CN.

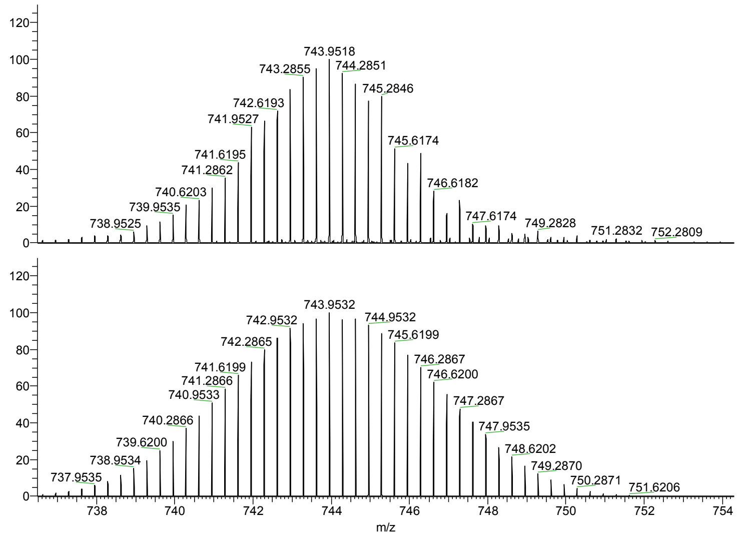

Figure S6. Comparison of experimental (upper trace) and calculated (lower trace) isotopic peaks for the ion $[PMo_{11}O_{39}SnC_{25}H_{26}BF_2N_2]^{3-}$ of $\mathbf{K^{Mo}_{Sn}[BOD_2]}$.

Differential pulse voltammetry

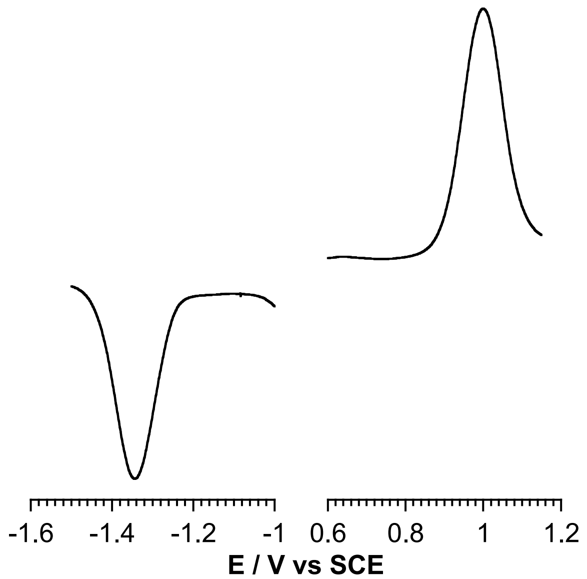

Figure S7. Differential pulse voltammograms of **BOD$_1$-TMS** in DCM containing 0.1 M TBAPF$_6$.

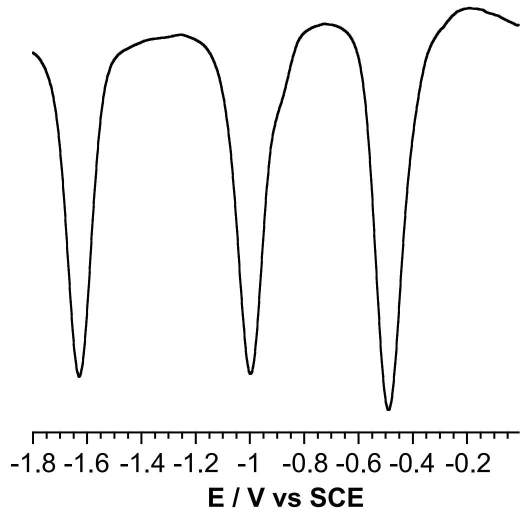

Figure S8. Differential pulse voltammograms of **K^{W}$_{Si}$[I]** in DCM containing 0.1 M TBAPF$_6$.

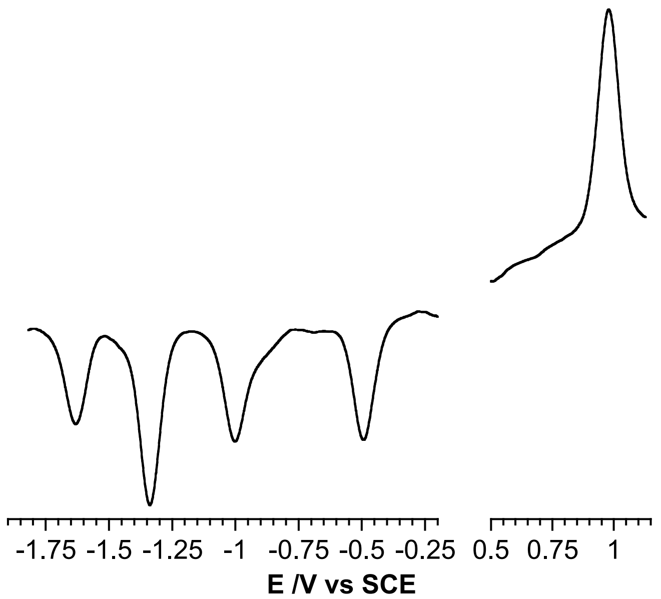

Figure S9. Differential pulse voltammograms of $\mathbf{K^{W}_{Si}[BOD_2]}$ in DCM containing 0.1 M $TBAPF_6$.

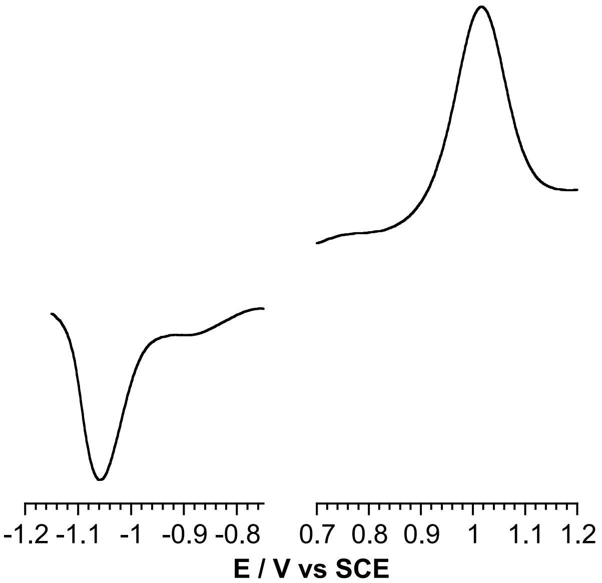

Figure S10. Differential pulse voltammograms of $\mathbf{BOD_2}$**-TMS** in DCM containing 0.1 M $TBAPF_6$.

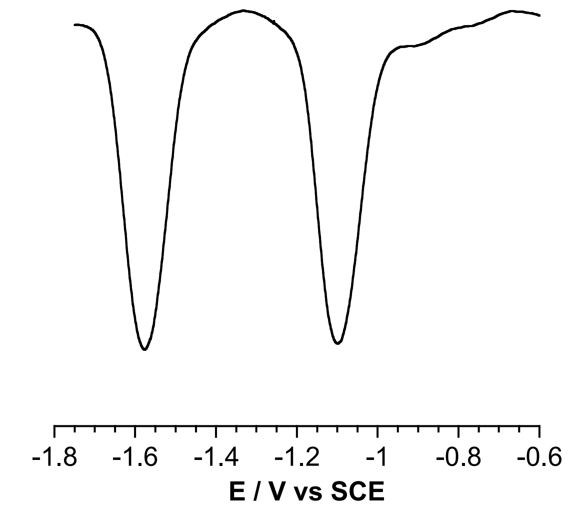

Figure S11. Differential pulse voltammograms of $\mathbf{K^{W}_{Sn}[I]}$ in DCM containing 0.1 M $TBAPF_6$.

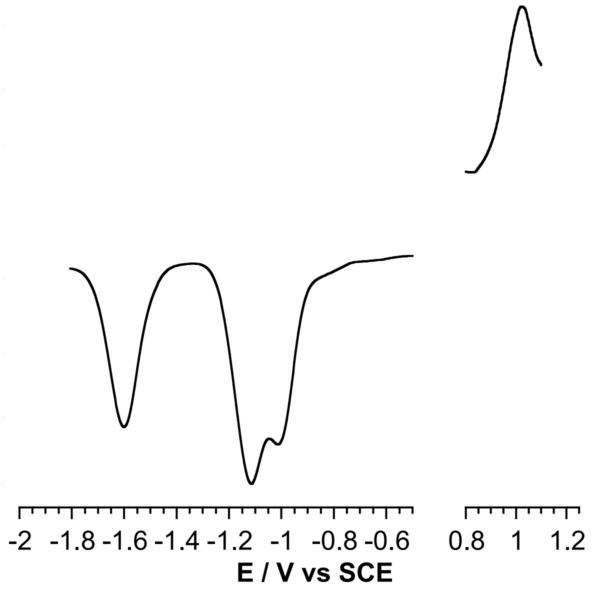

Figure S12. Differential pulse voltammograms of $\mathbf{K^{W}_{Sn}[BOD_2]}$ in DCM containing 0.1 M $TBAPF_6$.

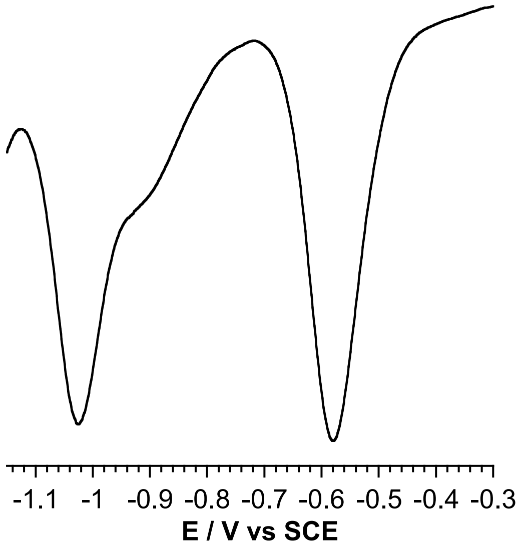

Figure S13. Differential pulse voltammograms of **K^{Mo}$_{Sn}$[I]** in DCM containing 0.1 M $TBAPF_6$.

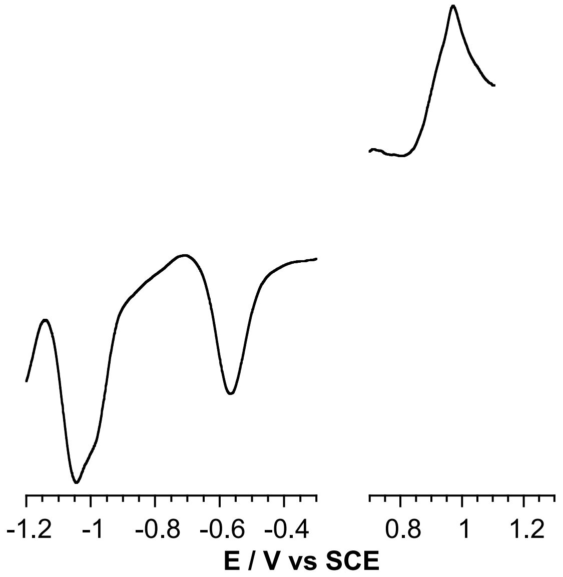

Figure S14. Differential pulse voltammograms of **K^{Mo}$_{Sn}$[BOD$_2$]** in DCM containing 0.1 M $TBAPF_6$.

Spectroelectrochemistry

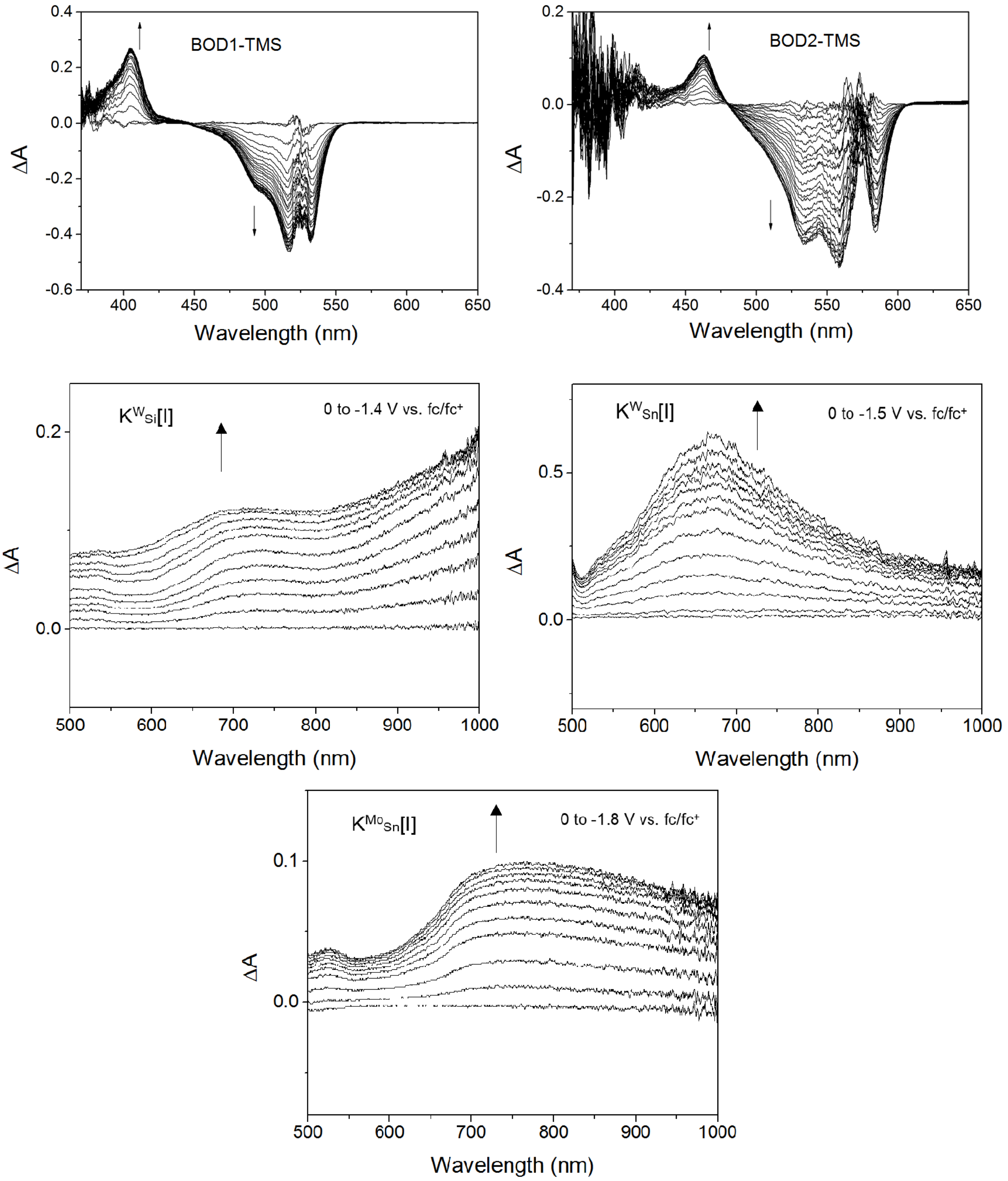

Figure S15. Absorption difference spectra of **BOD$_1$-TMS**, **BOD$_2$-TMS**, **K^{W}$_{Si}$[I]**, **K^{W}$_{Sn}$[I]** and **K^{Mo}$_{Sn}$[I]** in 0.2 M TBAPF$_6$ DCM solution showing the evolution of the oxidised bodipys and reduced POMs generated electrochemically.

Transient absorption spectroscopy

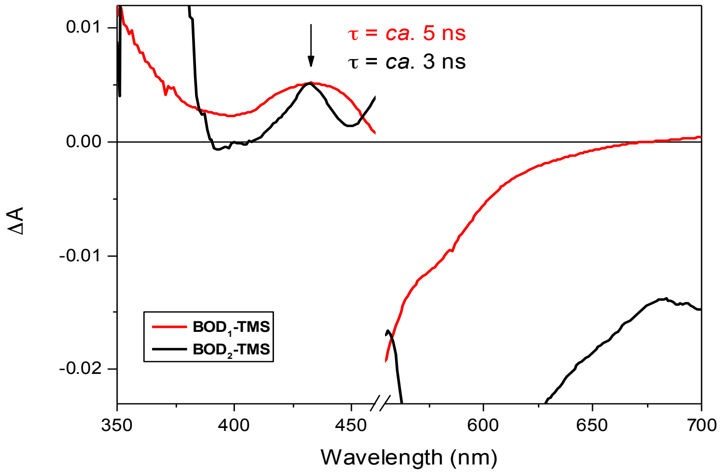

Figure S16. Ultrafast transient absorption spectra of **BOD$_1$-TMS** (red) and **BOD$_2$-TMS** (black) in DCM solution, 50 ps after excitation at 540 nm.

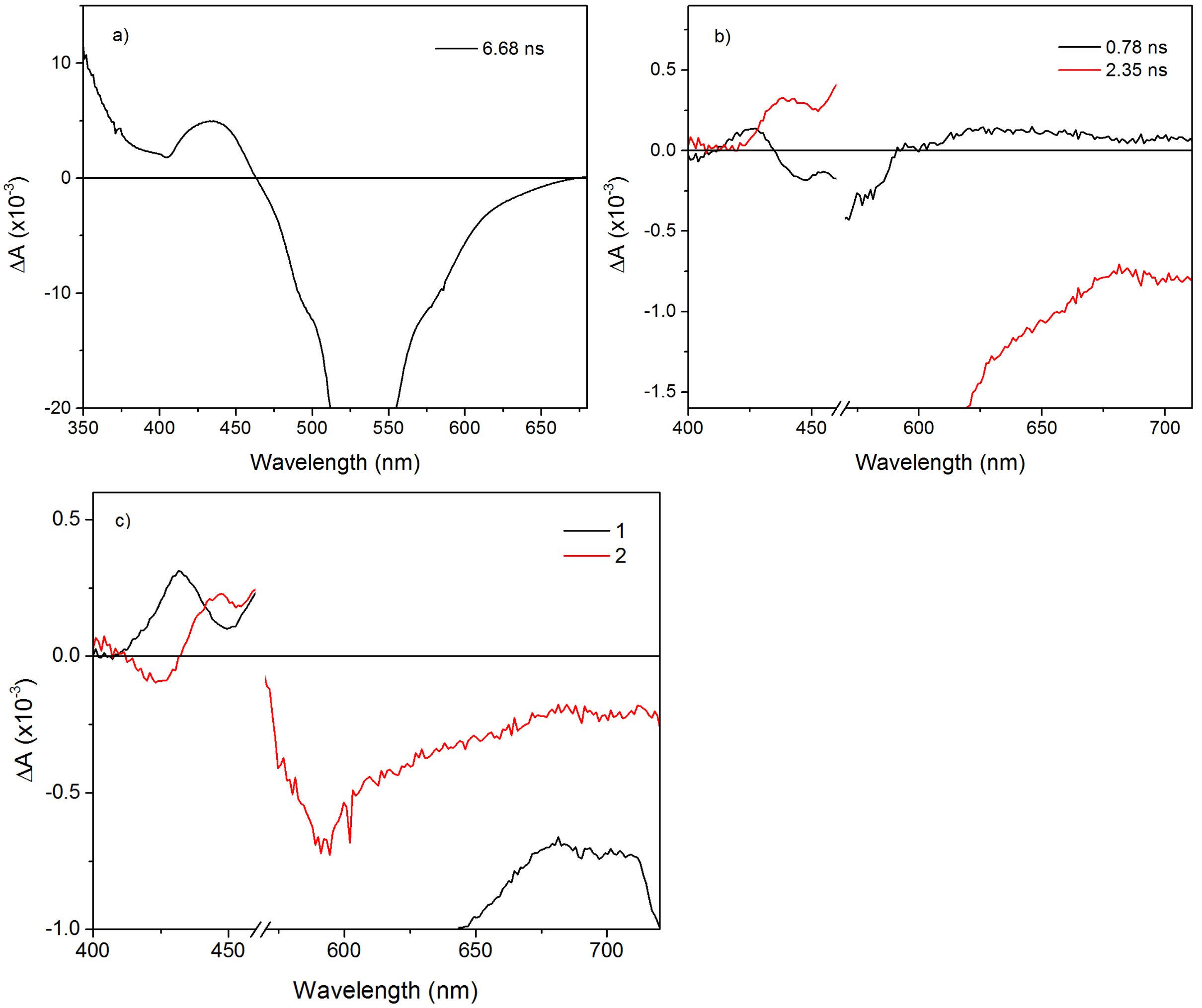

Figure S17. Decay associated difference spectra (DAS) of **BOD$_1$-TMS** (a) and **BOD$_2$-TMS** (b) in DCM solution. The DAS of **BOD$_1$-TMS** contains one species with a lifetime of 6.68 ns, consistent with the excited state. The DAS of **BOD$_2$-TMS** contains two species. The first

has a lifetime of 0.78 ns and the second, which is red-shifted relative to the first component, has a lifetime of 2.35 ns. (c) is the evolution associated difference spectra (EAS) of **BOD_2-TMS** in dichloromethane. A red shift of the excited state absorption is attributed to structural relaxation or photodecomposition of the sample (potentially the C-Si bond), consistent with the observed irreversible electrochemical oxidation (Figure 1).

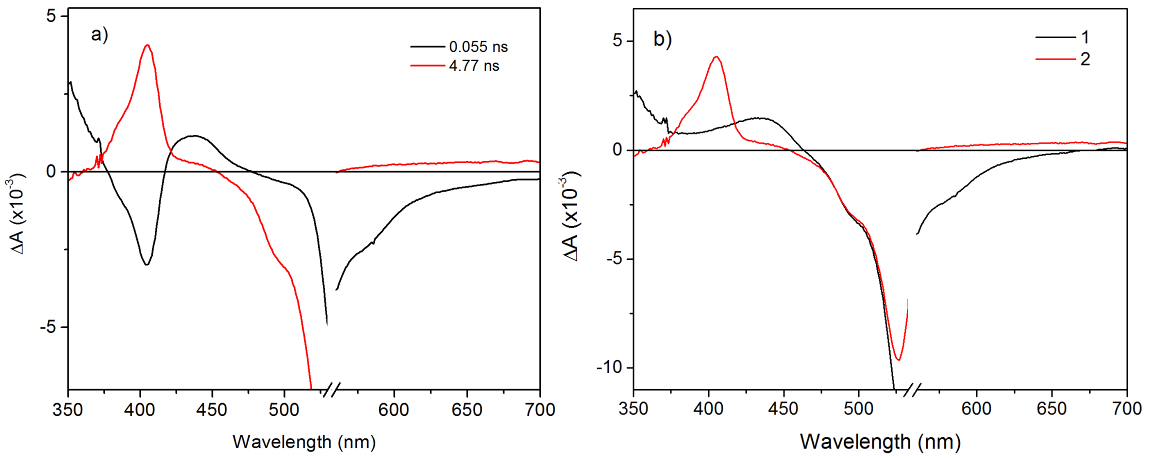

Figure S18. The decay associated difference spectra (a) and evolution associated difference spectra (b) of **K^{W}_{Si}[BOD_1]** in dichloromethane. The presence of negative amplitudes in the DAS is indicative of an excited state reaction. Component 1 (black), with a lifetime of 55 ps, resembles the excited state spectrum of **BOD_1-TMS**. Component 2 (red), with a lifetime of 4.77 ns, contains positive bands at 375-425 nm, consistent with the spectrum of the oxidised **BOD_1-TMS**, and above 550 nm, consistent with the spectrum of the reduced **K^{W}_{Si}[I]**, shown in the spectroelectrochemical analysis in Figure S15. This agrees with our single-wavelength analysis of the transient absorption spectra of **K^{W}_{Si}[BOD_1]** where we extracted an excited state lifetime of *ca*. 45 ps and a charge-separated state lifetime of *ca.* 3 ns.

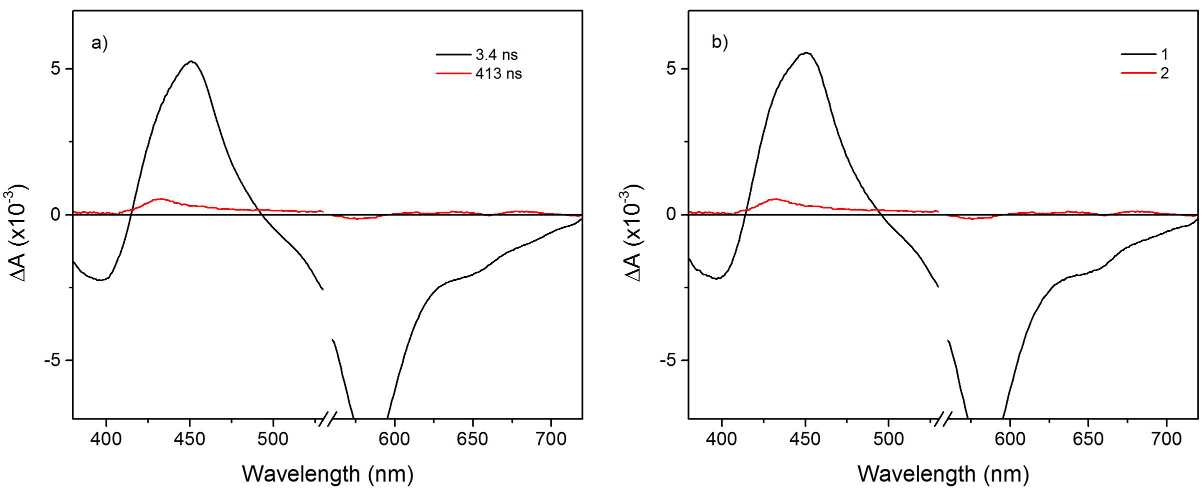

Figure S19. The decay associated difference spectra (a) and evolution associated difference spectra (b) of **K^{W}_{Sn}[BOD_2]** in dichloromethane. Component 1 (black) corresponds to the excited state absorption, ground state bleach and stimulated emission, consistent with decay from the bodipy excited state and the lifetimes are consistent with those determined from single wavelengths. A small second component (2), with a lifetime of 413 ns, is attributed to a small amount of the triplet state resulting from spin-orbit coupling with the POM.

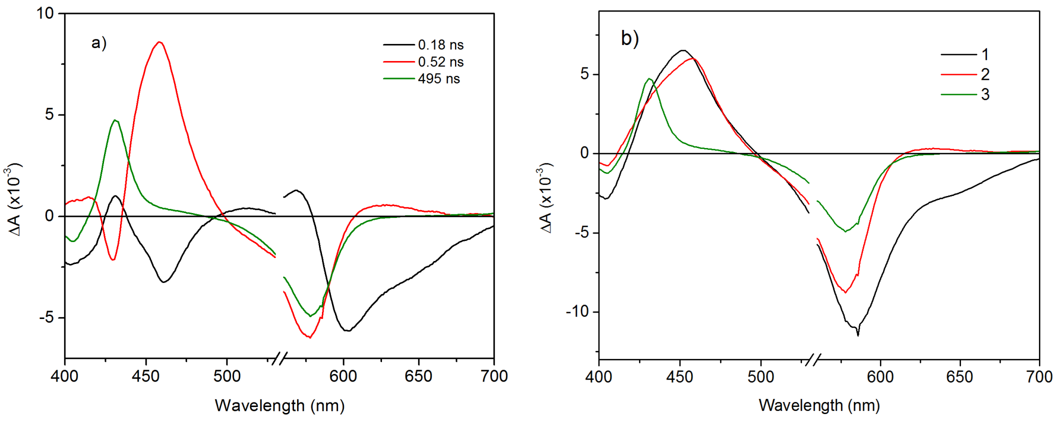

Figure S20. The decay associated difference spectra (a) and evolution associated difference spectra (b) of **$K^{Mo}{}_{Sn}$[BOD$_2$]**. Three components were extracted with lifetimes broadly consistent with those determined from single wavelengths. The first component (τ_1 = 180 ps) in the EAS has the same general shape as the **BOD$_2$-TMS** excited state, and contains a negative signal from the stimulated emission. The second component (τ_2 = 520 ps) is red-shifted (by 6 nm) relative to 1 and contains the ground state bleach but no stimulated emission. We have assigned this to the charge-transfer excited state, which decays to form the long-lived component (τ_3 = 495ns), which corresponds with the bodipy triplet excited state.

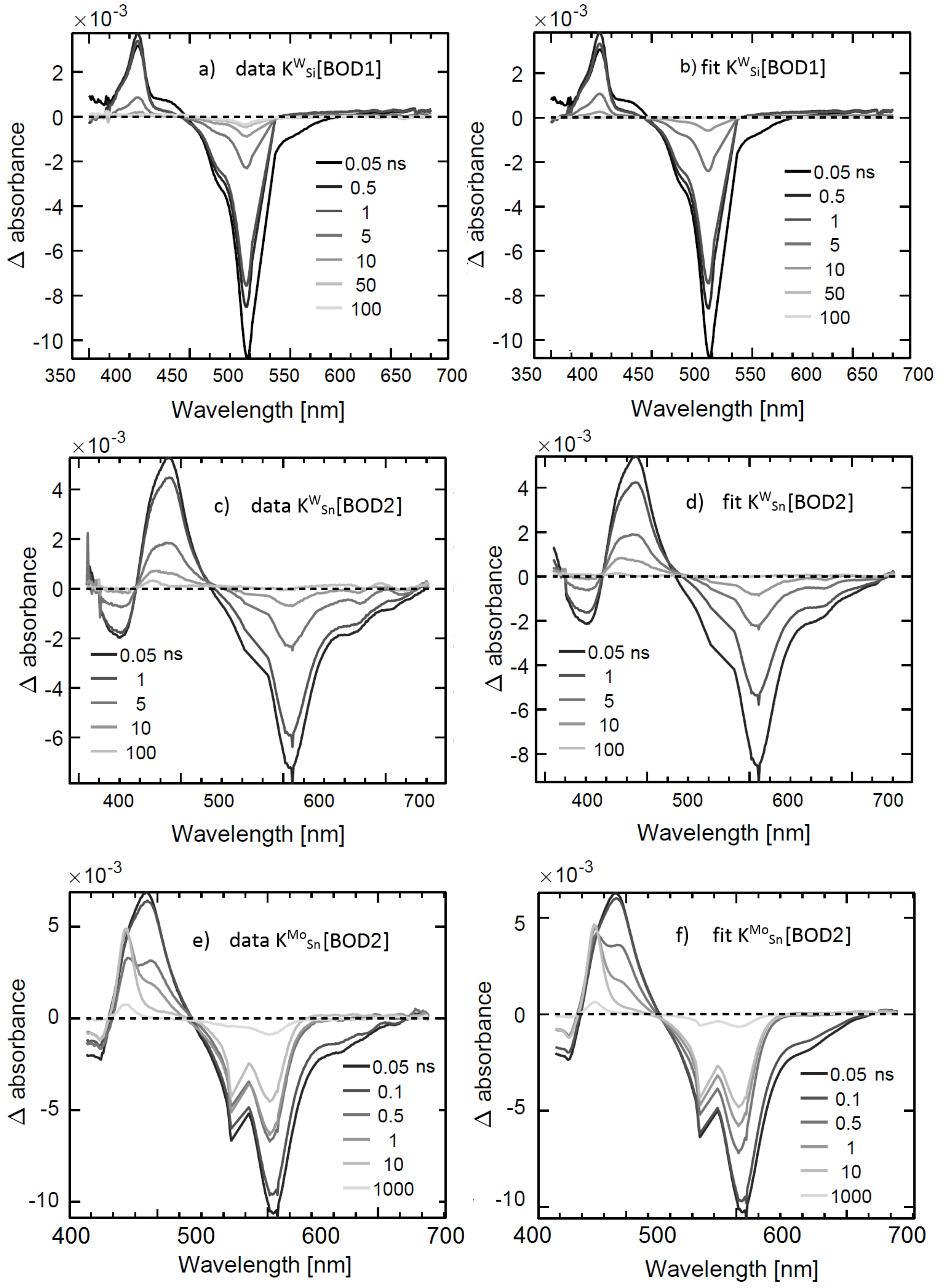

Figure S21. Comparison of experimental data with model extracted from the global analysis.

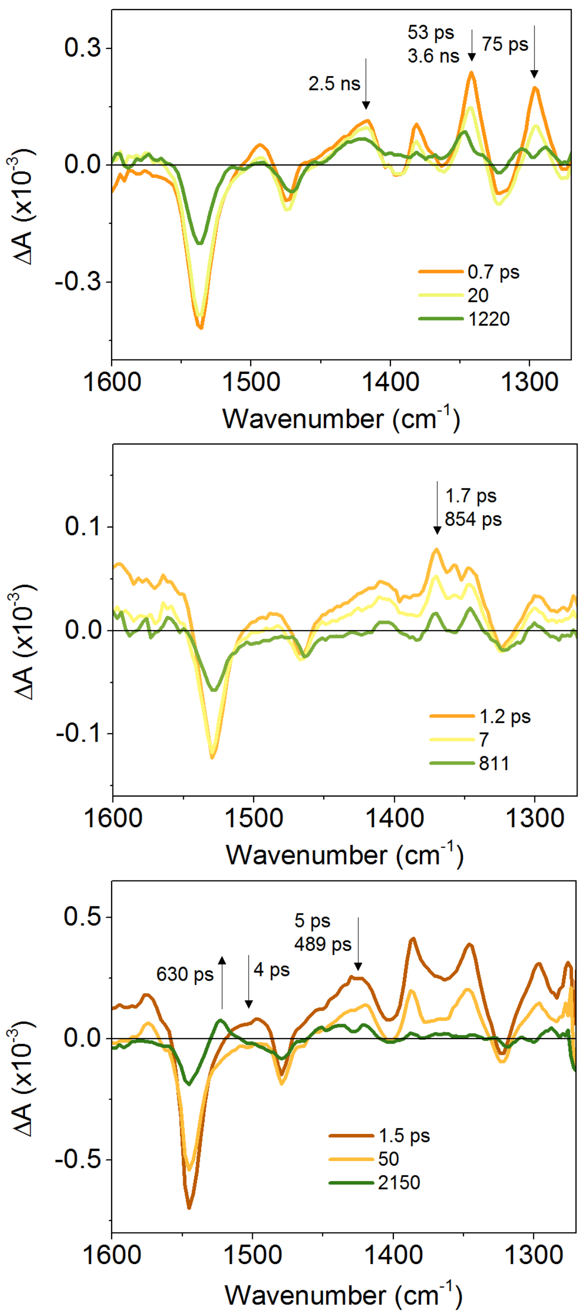

Figure S22. TRIR of POM-bodipy hydrids in DCM. Top: $\mathbf{K^{W}_{Si}[BOD_1]}$, middle: $\mathbf{K^{W}_{Sn}[BOD_2]}$, bottom: $\mathbf{K^{Mo}_{Sn}[BOD_2]}$.

Energy level diagram

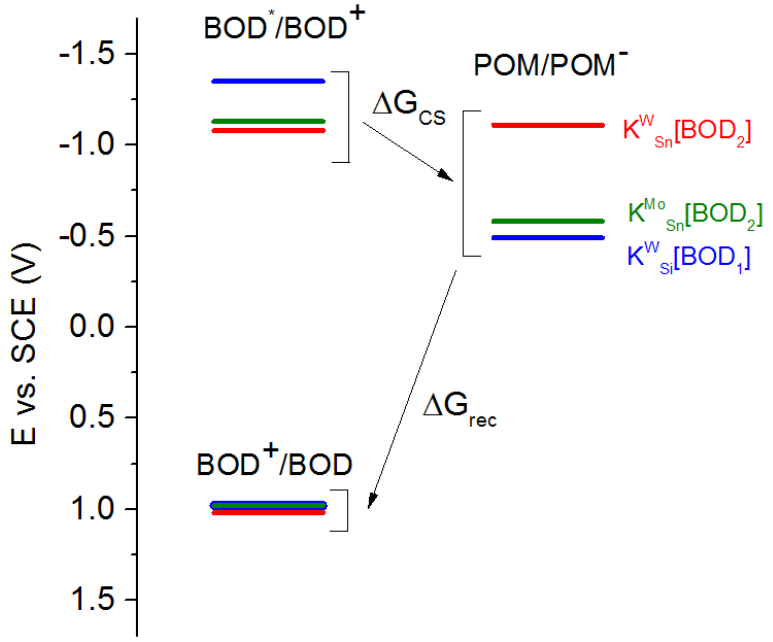

Figure S23. Energy level diagram of POM-bodipy hybrids in dichloromethane.

References

1. G. Ulrich and R. Ziessel, *J. Org. Chem.*, 2004, **69**, 2070-2083.
2. L. Bonardi, G. Ulrich and R. Ziessel, *Org. Lett.*, 2008, **10**, 2183-2186.
3. V. Duffort, R. Thouvenot, C. Afonso, G. Izzet and A. Proust, *Chem. Commun.*, 2009, DOI: 10.1039/b913475a, 6062-6064.
4. B. Matt, J. Moussa, L. M. Chamoreau, C. Afonso, A. Proust, H. Amouri and G. Izzet, *Organometallics*, 2012, **31**, 35-38.
5. C. Rinfray, S. Renaudineau, G. Izzet and A. Proust, *Chem. Commun.*, 2014, **50**, 8575-8577.
6. O. Dangles, F. Guibe, G. Balavoine, S. Lavielle and A. Marquet, *J. Org. Chem.*, 1987, **52**, 4984-4993.
7. G. M. Greetham, D. Sole, I. P. Clark, A. W. Parker, M. R. Pollard and M. Towrie, *Rev. Sci. Instrum.*, 2012, **83**.
8. C. Slavov, H. Hartmann and J. Wachtveitl, *Anal. Chem.*, 2015, **87**, 2328-2336.
